# Bioelectrochemically Powered Out‐of‐Equilibrium Hydrogels Enable Feedback‐Regulated Functions

**DOI:** 10.1002/advs.77934

**Published:** 2026-09-27

**Authors:** Roberto Baretta, Marco Frasconi

**Affiliations:** ^1^ Department of Chemical Sciences University of Padova Padova Italy

**Keywords:** dissipative systems, drug delivery, electrochemistry, hydrogels, polyrotaxane

## Abstract

Biological systems sustain functions by continuously converting energy into regulated processes through coupled reaction networks and feedback mechanisms. Achieving such functionality in synthetic materials remains challenging, as most energy‐driven molecular assembly only forms transient structures and cannot sustain operations under continuous energy input. Here, we report a bioelectrochemically powered hydrogel that operates far‐from‐equilibrium via a fully electrochemical antagonistic redox cycle coupled to enzymatic feedback. We establish a fully electrochemical cycle in which reduced vitamin B_12_ and oxidized ferrocene mediate disulfide cleavage and reformation within a disulfide‐cross‐linked polyrotaxane‐based hydrogel, enabling dissipative growth and temporal modulation of mechanical properties. Embedding glucose oxidase within the hydrogel introduces a glucose‐dependent enzymatic pathway that competes for ferrocene against disulfide reformation, inducing partial network decrosslinking and increased permeability under high‐glucose level. This enzymatic feedback mechanism enables a glucose‐responsive insulin delivery platform with enhanced release at high glucose and suppressed release at low glucose. These findings establish a general strategy to couple electrochemical energy input with biochemical feedback, enabling adaptive regulation of structure, mechanics and transport in soft materials under nonequilibrium conditions.

## Graphical abstract

A bioelectrochemically powered hydrogel operates far from equilibrium via an antagonistic redox cycle that regulates disulfide exchange within a polyrotaxane network. Coupling electrochemical mediator cycling with enzymatic feedback enables dynamic control of hydrogel mechanics and transport properties, driving glucose‐responsive insulin release with tunable dosage and timing, and establishing a general strategy for autonomous, adaptive soft materials.

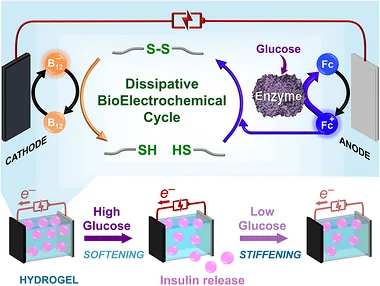

## 1 Introduction

Living systems operate far from thermodynamic equilibrium, sustaining structure and function through continuous energy consumption. Growth, adaptation, and regulation arise from antagonistic reaction networks and feedback loops that couple molecular transformations to larger‐scale control processes, enabling autonomous operations [1]. Translating these principles to synthetic materials would enable adaptive biointerfaces and biomedical devices [2, 3], including precision therapeutic systems capable of autonomously modulating drug delivery in response to physiological fluctuations [4]. However, achieving such integrated functionality under continuous energy input remains a major challenge for synthetic materials.

Dissipative assembly has emerged as a powerful strategy for developing adaptive soft materials, in which continuous energy input sustains dynamic structures under nonequilibrium conditions [5, 6, 7, 8]. Chemically fueled reaction cycles [9, 10, 11, 12, 13], light‐driven processes [14, 15, 16], mechanically induced transformations [17], and electrochemically mediated redox reactions [18, 19] have all been used to generate and maintain such assemblies through continuous energy consumption. Electricity is particularly attractive as energy input because it is clean, spatially addressable, temporally programmable, and readily integrated into devices [20, 21, 22, 23]. Furthermore, electrons supplied by an electrode under an applied potential can act directly as reagents, enabling spatially confined material growth [24, 25]. In the context of dissipative systems, electrochemically activated redox mediators have been employed to drive transient supramolecular assembly at electrode interfaces [18]. Dissipative growth of disulfide‐crosslinked hydrogels has been achieved under biocompatible conditions through electrochemical generation of oxidizing mediators that convert thiols into disulfide cross‐links in the presence of a chemical reductant in solution [26]. Electrochemical inputs have likewise enabled transient modulation of the mechanical properties of hydrogel films at electrode interfaces [27, 28]. A compelling direction is the development of fully electrochemical dissipative systems in which both activating and deactivating redox species are generated in situ, establishing a redox cycle that sustains the transient process under constant bias without the consumption of sacrificial reagents. For example, electrochemical oxidation of ferrocyanide and reduction of methyl viologen, which serve as complementary redox mediators, have been reported to enable reversible assembly and disassembly of fibers from cysteine‐derived building blocks [29]. Despite these advances, most dissipative materials remain confined to transient assembly, where structures assemble under energy input and disassemble upon its removal but rarely exhibit energy‐driven, dynamically regulated functions. Particularly, in hydrogel networks, performing operations far from equilibrium can lead to mechanical fatigue or uncontrolled depolymerization unless the structure enables dynamic reorganization without collapse [30, 31]. Achieving dissipative autonomous operations therefore requires coupling continuous energy input with structural reconfiguration and feedback regulation, while preventing network collapse under multiple cycling.

**FIGURE 1:**
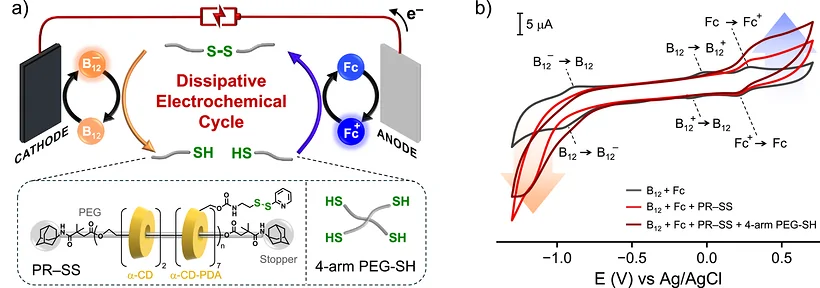
Fully electrochemically driven growth of hydrogels. (a) Schematic representation of the electrochemically sustained antagonistic redox cycle, where reduced vitamin B_12_ mediates disulfide cleavage, and oxidized ferrocene restores disulfide bonds. The hydrogel, composed of disulfide‐functionalized polyrotaxane (PR–SS) and 4‐arm PEG–SH, grows from the cathode. (b) Cyclic voltammograms (250 mV/s) recorded in 0.1 M PBS solution (pH 7.4) under inert atmosphere containing 100 µM B_12_ and 50 µM Fc (black curve), upon addition of 50 µM PR–SS (red curve), and upon addition of 50 µM PR–SS with 100 µM 4‐arm PEG‐SH (dark red curve).

Here we report a bioelectrochemically powered hydrogel in which an electrochemically sustained antagonistic redox cycle, coupled to enzymatic feedback, dynamically regulates network structure and functions. The system integrates a disulfide‐functionalized polyrotaxane with a 4‐arm thiol‐terminated polyethylene glycol to form a dynamically crosslinked network governed by voltage‐controlled redox cycle mediated by vitamin B_12_ and ferrocene carboxylic acid (Figure 1a). Cathodically generated reduced vitamin B_12_ cleaves disulfide bonds, whereas anodically generated ferricenium (Fc^+^) promotes thiol re‐oxidation to disulfide. Together, these electrochemically coupled processes establish an antagonistic reaction network in which disulfide cleavage and reformation occur simultaneously, enabling dissipative hydrogel growth with dynamic tunability of crosslink density, composition, and stability. Continuous electrochemical regeneration of both oxidant and reductant sustains the hydrogel in a dynamic nonequilibrium state powered exclusively by electrical energy, allowing continuous network rearrangement without consumption of chemical reagents or accumulation of waste. Polyrotaxane incorporation, through its mechanically interlocked architecture [32, 33], improves the mechanical stability of the hydrogel network during redox cycling. The resulting networks exhibit high cross‐link density, enhanced viscoelastic tolerance and robustness, enabling stress redistribution during continuous disulfide exchange and preventing mechanical failure under out‐of‐equilibrium conditions. Building on this platform, we integrate glucose oxidase within the hydrogel to directly couple enzymatic activity to the antagonistic redox cycle. Glucose‐dependent enzymatic turnover introduces a competing pathway for Fc^+^ consumption, limiting its availability for thiol oxidation and shifting the redox balance toward disulfide cleavage, softening the network and enhancing insulin release; as glucose decreases, oxidative recrosslinking tightens the network and suppresses release. In contrast to existing glucose‐responsive insulin delivery strategies that rely on irreversible swelling or single‐trigger activation [34], this system enables electrically sustained, dynamically regulated insulin release in response to fluctuations within the physiological glucose range. This work establishes a design principle for bioelectrochemically sustained dissipative soft materials in which electrochemical energy input and enzymatic feedback jointly regulate structure and functions under out‐of‐equilibrium conditions.

## 2 Results and Discussion

### 2.1 Fully Electrochemical Dissipative Growth of Hydrogel Films With Tunable Properties

To develop a mechanically robust dissipative platform, we designed a disulfide‐crosslinked hydrogel composed of 4‐arm thiol‐terminated polyethylene glycol (4‐arm PEG–SH) and a disulfide‐functionalized polyrotaxane (PR–SS). Incorporation of the polyrotaxane was expected to enhance network stability by providing a high density of cross‐linkable disulfide sites while preserving deformability through mobile junctions that redistribute stress during redox cycling, thereby preventing network failure. The polyrotaxane was synthesized by threading α‐cyclodextrins (CD) onto a polyethylene glycol backbone (PEG, 10 kDa), followed by end‐capping with adamantyl groups to prevent dethreading [35, 36], resulting in an average of 9 CD rings per chain. Functionalization of the threaded CD units with 2‐(pyridyldithio)ethylamine (PDA) introduced disulfide moieties along the scaffold [37], yielding a disulfide‐functionalized polyrotaxane (Figure 1a) bearing approximately 7 PDA side chains per polyrotaxane, as determined by ^1^H NMR spectroscopy (Figures S1–S7). To establish a fully electrochemical dissipative platform for the growth and sustained operation of disulfide‐crosslinked hydrogel films, we identified two redox mediators operating at sufficiently separated potentials in aqueous solution to support independent electrocatalytic cycles compatible with the disulfide network. Ferrocene carboxylic acid (Fc) was employed as the redox mediator for the anodic cycle owing to its favorable kinetics for thiol oxidation in polymeric hydrogels under mild aqueous conditions while maintaining biocompatibility in the presence of enzymes and even living cells [38, 39].

For the cathodic cycle, vitamin B_12_ (B_12_) was used as the mediator, based on its redox potential, biocompatibility, and established electrocatalytic activity toward disulfide reduction to thiols in aqueous media [40]. To verify the electrochemical compatibility of the two mediators, cyclic voltammetry (CV) was performed on a solution containing 100 µM B_12_ and 50 µM Fc in 0.1 M phosphate‐buffered saline (PBS) at pH 7.4 under nitrogen. The voltammogram shows two well‐defined redox couples for B_12_, corresponding to B_12_ Co(II) / B_12_^−^ Co(I) at −970 mV and −805 mV, and B_12_^+^ Co(III) / B_12_ Co(II) at −117 and −25 mV (Figure 1b). A distinct redox couple for Fc (Fc^+^/Fc) is observed at 221 and 301 mV. These responses closely match those of the individual mediators recorded separately (Figure S10), and the cathodic‐to‐anodic peak current ratios for both B_12_ and Fc remain close to unity, indicating reversible behavior with no electrochemical interference between the two mediators. Upon addition of 50 µM PR–SS to the solution, the CV responses of the mediators change markedly. An increase in the cathodic current accompanied by attenuation of the corresponding anodic peak is observed for B_12_ / B_12_^−^ couple, consistent with electrocatalytic reduction of disulfide groups on PR–SS to thiols by the reduced B_12_ species. Conversely, Fc shows a pronounced enhancement of the anodic current accompanied by suppression of the corresponding cathodic peak, indicating that Fc^+^ mediates reoxidation of the generated thiols back to disulfides. When the CV scan is initiated from 0 V toward positive potentials, no catalytic current is observed at the Fc anodic peak, indicating that reduction of disulfide groups on PR–SS precedes Fc‐mediated thiol oxidation (Figure S11). A further increase in the catalytic anodic current of Fc can be observed upon addition of 4‐arm PEG–SH (100 µM), which supplies additional thiols that participate in Fc^+^‐mediated formation of disulfide cross‐links. To gain further mechanistic insight into the antagonistic redox cycles, we determined the homogeneous electron‐transfer rate constant (*k_et_*) for B_12_‐mediated disulfide reduction on PR–SS (Figure S12), obtaining a value of (3.6 ± 0.5) × 10^4^ M^−1^ s^−1^. This high rate constant indicates rapid electrocatalytic cleavage of disulfide bonds by the reduced B_12_ species. For comparison, the *k_et_* for Fc‐mediated thiol oxidation to disulfide is 450 M^−1^ s^−1^ [26], indicating that the reductive pathway proceeds faster than the oxidative one. The difference in *k_et_* values establishes distinct kinetic timescales for disulfide cleavage and reformation, which influence the transient evolution of cross‐link density under applied voltage.

Having established the electrochemical compatibility of the mediators and the kinetic asymmetry of the antagonistic redox cycles by cyclic voltammetry, we next examined how this system enables the growth of disulfide‐crosslinked hydrogel films. The fully electrochemical dissipative growth of the hydrogel films was carried out under chronoamperometric conditions using a parallel‐plate electrochemical cell (Figure S13) consisting of a platinum counter electrode and a screen‐printed electrode comprising a glassy carbon working electrode and an Ag/AgCl reference electrode. The working and counter electrodes were separated by 2 mm and immersed in a degassed 0.1 M PBS solution (pH 7.4) containing 0.5 mM B_12_, 4 mM Fc, 1.5 mM PR–SS, and 1 mM 4‐arm PEG–SH. Because reduction of the disulfide groups on PR–SS is required to generate thiol groups that subsequently undergo oxidative cross‐linking with 4‐arm PEG–SH, constant cathodic potentials of −0.8, −1.0, or −1.2 V vs. Ag/AgCl were applied to the working electrode. These potentials were selected based on the CV experiments, which identified the onset to promote the B_12_‐mediated reduction process (Figure 1b), while avoiding the more extensive water electrolysis expected at higher cathodic potentials. Consequently, hydrogel growth at the cathode proceeds through sequential reductive activation of PR–SS followed by Fc^+^‐mediated oxidative disulfide cross‐linking sustained by the diffusion of Fc^+^ from the anode. Upon applying −0.8 V, the hydrogel film reached a maximum thickness of 178 µm after 20 min, followed by a gradual decrease at longer times (Figure S14). Applying more negative potentials, −1.0 and −1.2 V, accelerates the formation of the film at the cathode but also promotes more rapid hydrogel degradation. Notably, hydrogel films form only in the presence of both mediators, as no films are observed when a negative potential is applied to solutions containing either Fc or B_12_ alone. These results indicate that film growth is transient and governed by the voltage‐controlled antagonistic redox cycle that drives reductive decrosslinking and oxidative recrosslinking of disulfide bonds through the diffusion of redox mediators. Furthermore, hydrogel growth strongly depends on the interelectrode distance. With an interelectrode distance of 1 mm, the film reached a thickness of 65 µm, which increased upon increasing the separation between the cathode and anode to 2 mm. A further increase of interelectrode distance from 2 to 4 mm progressively reduced the hydrogel thickness (Figure S16), highlighting the critical role of mediator diffusion and the spatial overlap between the reductive and oxidative reaction fronts.

**FIGURE 2:**
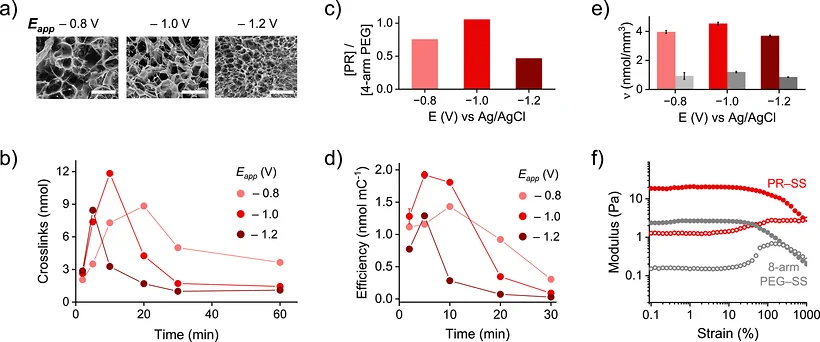
Transient growth of hydrogel films. (a) SEM images of hydrogel films formed from PR‐SS and 4‐arm PEG‐SH in the presence of Fc and B_12_ at different applied potentials (scale bar: 50 µm). (b) Disulfide crosslinks as a function of the deposition time for hydrogel films obtained at different applied potentials (V): −0.8, −1.0 and −1.2. (c) Ratio between PR and 4‐arm PEG in the hydrogel network as a function of the applied potential for films obtained at maximum crosslinks. (d) Electrochemical deposition efficiency of hydrogel films as a function of deposition time under different applied potentials. (e) Cross‐link density (ν) as a function of applied potential for hydrogel films obtained under dissipative conditions from PR–SS (red) or 8‐arm PEG–SS (gray). (f) Strain sweeps (1 Hz) of hydrogels films obtained from PR–SS (red circles) and 8‐arm PEG–SS (gray circles) at −1.0 V applied potential for 10 min (*G’* full circles, *G″* hollow circles). Hydrogel films were prepared from solution containing PR–SS (1.5 mM) or 8‐arm PEG–SS (1.3 mM) and 4‐arm PEG–SH (1 mM) in the presence of Fc (4 mM) and B_12_ (0.5 mM) at the indicated applied potential.

Scanning electron microscopy (SEM) of the freeze‐dried hydrogel films revealed a porous morphology characteristic of hydrogels (Figure 2a), with the pore diameter progressively decreasing as the applied potential became more negative. Cross‐sectional SEM images showed no appreciable variation in the micrometer‐scale porous morphology from the electrode interface to the outer film surface exposed to the solution under the investigated deposition conditions (Figure S15). Because SEM analysis requires freeze‐drying of the hydrogel films, the observed pore morphology does not directly reflect the molecular mesh size of the swollen hydrogel network. To confirm that the hydrogel films are cross‐linked by disulfide bonds, the hydrogel‐coated electrodes were immersed in a solution of the reducing agent tris(2‐carboxyethyl)phosphine hydrochloride (TCEP) for 1 h. This treatment resulted in complete dissolution of the films, confirming disulfide cross‐linking within the network. The total thiol content, and thus the polymer amount deposited on the electrode, was determined by a colorimetric assay [41] after dissolving hydrogel films prepared at different deposition times. At all applied potentials, the thiol content exhibits transient behavior, initially increasing upon voltage application, reaching a maximum, and then decreasing at longer times until near‐complete film dissolution occurs after 60 min. The applied potential strongly affects the time‐dependent growth of hydrogel films. As the applied potential becomes more negative, the maximum polymer deposition is reached at shorter times. The amount of deposited polymer at the maximum, expressed through the number of disulfide crosslinks, is 8.8 ± 0.2 nmol at −0.8 V after 20 min, 11.8 ± 0.2 nmol at −1.0 V after 10 min, and 8.4 ± 0.1 nmol at −1.2 V after only 5 min (Figure 2b). This transient growth of the hydrogel arises from the dissipative nature of the process, in which continuous electrochemical energy input sustains an antagonistic redox cycle for continuously driving disulfide bond cleavage and reformation, causing the hydrogel to grow, reaching a maximum thickness, and ultimately undergoing complete depolymerization upon prolonged voltage application. The ratio between PR and 4‐arm PEG in those networks is approximately 1 for films deposited at −1.0 V for 10 min (Figure 2c), whereas lower PR contents are observed at other applied voltages, as determined by ^1^H NMR spectroscopy of the dissolved films (Figure S18). In addition, progressive decrease in the PR:4‐arm PEG ratio is observed over time (Figure S19). These results suggest that B_12_‐mediated cleavage of disulfides groups on PR–SS generates reactive thiol groups on the polyrotaxane, thereby activating the precursor for subsequent Fc^+^‐mediated oxidative cross‐linking with 4‐arm PEG to form the hydrogel network. Following the initial film formation, the continuous operation of the redox cycle dynamically remodels the hydrogel through repeated disulfide cleavage and reformation. As a result, the film progressively decreases in thickness and cross‐link density, while the network becomes relatively enriched in the smaller 4‐arm PEG component. The presence of both 4‐arm PEG and PR is essential for film formation. No film is obtained when electrodeposition is performed with PR–SS as the only monomer, suggesting that direct cross‐linking between PR molecules is unfavorable, likely due to steric hindrance from the short thiol side chains grafted onto the bulky CD rings. Likewise, no film forms at the cathode when a negative potential is applied to a solution containing only 4‐arm PEG. As a control, electrodeposition was performed in the absence of B_12_ by applying an oxidative potential to promote disulfide formation under equilibrium conditions. Under these conditions, weak hydrogel films formed, consisting exclusively of 4‐arm PEG, as confirmed by ^1^H NMR analysis (Figure S18H), and exhibiting constant cross‐link density over time (Figure S20).

These results highlight the essential role of B_12_ in the dissipative growth process, enabling incorporation of PR–SS into the hydrogel matrix and dynamic modulation of network cross‐linking through controlled disulfide bond cleavage. The different growth behaviors at varying potentials arise from increased electrochemical generation of the oxidized Fc and reduced B_12_, which simultaneously promote cross‐linking of the soluble polymer precursors and bond exchange within the deposited hydrogel, thereby governing the balance between film growth and network remodeling. Application of more negative potentials at the cathode promote the reduction of larger amounts of B_12_, and the oxidation of increasing amounts of Fc at the anode (Figure S17). At −0.8 V, limited reduction of PR–SS delays film growth and leads to enrichment of 4‐arm PEG in the film (Figure 2c). At −1.0 V, film formation is faster and results in enrichment of PR. At −1.2 V, however, reduction of the disulfide‐cross‐linked network becomes predominant over its formation, resulting in rapid film degradation. The maximum efficiency of dissipative growth, calculated from the ratio between the formed cross‐links and the total charge consumed during deposition, is obtained at −1.0 V for deposition times of 5–10 min (Figure 2d). It is important to note that the electrochemically generated mediators can react with both the soluble polymer precursors and the deposited hydrogel network. Consequently, charge is consumed not only to promote hydrogel growth through oxidative cross‐linking of polymers, but also to drive bond exchange and network remodeling within the deposited film, as well as by competing reactions in the surrounding solution. The dependence of hydrogel growth on the applied potential and deposition time indicates that reactions occurring in the vicinity of both the electrode and the growing hydrogel dominate the assembly process.

To evaluate the influence of the polyrotaxane architecture on the properties of the resulting hydrogels, a non‐mechanically interlocked polymer was employed as a control. Specifically, a branched thiolated 8‐arm PEG, matching the molecular weight of the PEG axle in PR and bearing an equivalent number of cross‐linking sites, was functionalized with disulfide units, as demonstrated by ^1^H and ^13^C NMR spectroscopy (8‐arm PEG–SS, Figures S8 and S9). Hydrogel films were electrodeposited from solutions containing 0.5 mM B_12_, 4 mM Fc, 1 mM 4‐arm PEG‐SH, and 8‐arm PEG–SS (1.3 mM), which provides the same total concentration of disulfide groups as PR–SS. Applying −0.8 V for 20 min, −1.0 V for 10 min, and −1.2 V for 5 min yields hydrogel films with thickness comparable to PR–SS‐based systems under the same conditions (Figure S21), but with cross‐link densities about four times lower (Figure 2e). These results indicate that incorporation of the polyrotaxane promotes formation of more highly reticulated networks, likely due to the sliding motion of the rings along the PEG axle that enables network reorganization during transient deposition and enhances polymer incorporation. We next investigated the rheological properties of hydrogel films obtained from PR–SS and 8‐arm PEG–SS (Figures 2f and Figure S22). In all cases, storage modulus (*G’*) exceeds loss modulus (*G″*) over a wide range of strain and frequency, confirming the elastic nature of the networks. PR–SS‐based hydrogels display significantly higher *G’* values than 8‐arm PEG–SS films. For films deposited at −1.0 V, the PR–SS‐based hydrogel exhibits a *G’* of 20 ± 2 Pa, whereas the 8‐arm PEG–SS film shows only 2 ± 1 Pa, consistent with the higher cross‐link density of the PR–SS network. PR–SS hydrogels also maintain viscoelastic behavior over a wider strain range, with *G’* = *G″* occurring at approximately 1000% strain compared to 250% for 8‐arm PEG–SS films. This enhanced mechanical stability under deformation is attributed to additional energy dissipation from the sliding motion of the rings in the polyrotaxane network [35]. Overall, these results demonstrate that dissipative hydrogel growth is governed by an antagonistic redox cycle sustained by continuous electrical energy input, enabling precise control over the network properties through dynamic disulfide bond exchange.

### 2.2 Electrochemically Driven Dissipative Hydrogel Softening

**FIGURE 3:**
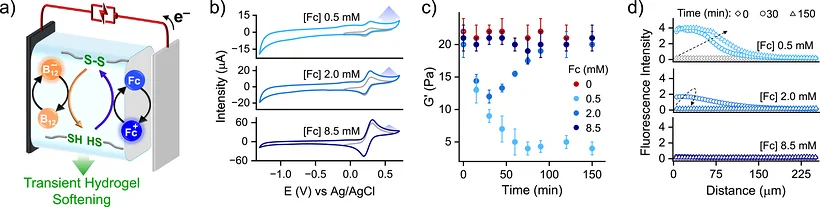
Fully electrochemical modulation of hydrogel mechanical properties. (a) Schematic representation of transient hydrogel softening under continuous voltage application, driven by disulfide bond cleavage mediated by cathodically generated B_12_^−^ and reformation of disulfide cross‐links mediated by anodically generated Fc^+^. (b) Cyclic voltammograms (10 mV s^−1^) recorded under inert atmosphere at working electrodes modified with PR‐based hydrogel films in 0.1 M PBS (pH 7.4) containing 50 µM B_12_ and increasing concentrations of Fc. Gray curves correspond to CVs recorded under identical conditions within the potential window −0.1 to 0.6 V. (c) Temporal changes in overall hydrogel stiffness (*G’*) under application of −1.0 V in a 50 µM B_12_ solution in PBS 0.1 M (pH 7.4) under inert atmosphere in the presence of increasing concentrations of Fc (mM): 0.5, 2.0 and 8.5. Red curve represents temporal changes in hydrogel stiffness under application of −1.0 V in the absence of both mediators. (d) Fluorescence intensity profiles as a function of distance from the electrode surface for hydrogels under continuous voltage application at different Fc concentrations (arrows indicate the temporal evolution).

Having established a fully electrochemical strategy for dissipative growth of hydrogel films, we next investigated how the applied potential modulates the mechanical properties of the pre‐formed hydrogel (Figure 3a). Under these conditions, the applied potential no longer drives hydrogel growth but instead sustains the antagonistic redox cycle within the hydrogel network, continuously promoting disulfide bond cleavage and reformation and thereby enabling dynamic network remodeling. For the following experiments, PR‐based hydrogel films were deposited at −1.0 V for 10 min, as they exhibit higher cross‐link density and enhanced network stability, and then investigated in different B_12_: Fc ratios, to probe the antagonistic behavior of the mediators and their effect on disulfide bond cleavage and reformation in the hydrogel network. Cyclic voltammetry (CV) was performed on glassy carbon (GC) electrodes modified with the hydrogel film in solutions containing 50 µM B_12_ and varying Fc concentrations (0.5, 2.0, and 8.5 mM) in 0.1 M PBS (pH 7.4). CV displays an electrocatalytic cathodic current for B_12_ corresponding to reduction of disulfide cross‐links in the hydrogel network. Upon reversing the scan toward oxidative potentials, an electrocatalytic anodic current for Fc is observed, indicating Fc‐mediated oxidation of the thiols generated by reduced B_12_ (Figure 3b). Increasing the Fc concentration leads to higher overall currents due to the greater concentration of redox‐active species, while the relative electrocatalytic enhancement decreases. This behavior indicates a transition from an electrode‐limited regime, where Fc^+^ is continuously regenerated at the electrode surface, to a mediator‐excess regime in which a high flux of Fc^+^ diffuses into the hydrogel and sustains thiol oxidation within the network, thereby diminishing the contribution of electrochemical regeneration to the overall catalytic process.

The hydrogel film in the parallel‐plate electrochemical cell was then subjected to a constant potential of −1.0 V in a solution containing 50 µM B_12_ and varying Fc concentrations, and the overall stiffness of the hydrogel deposited on the electrode was monitored by rheometry at defined time intervals (Figure 3c). At low Fc concentration (0.5 mM), *G’* progressively decreases from an initial value of 20 Pa to approximately 4 Pa after 60 min of applied potential. This decrease in stiffness is consistent with an overall network weakening caused by disulfide bond cleavage mediated by B_12_^−^ generated at the cathode. Simultaneously, Fc^+^ diffusing from the anode promotes thiol reoxidation, partially counterbalancing the network weakening. As a result, a dynamic steady state is established in which continuous disulfide bond cleavage and reformation maintain the hydrogel in a softened state far from equilibrium without complete disassembly from the electrode. Increasing the Fc concentration to 2 mM results in an initial decrease in stiffness, with *G’* reaching 12 Pa after 30 min, followed by a gradual recovery to its original value within approximately 90 min (Figure 3c). This behavior indicates that the hydrogel undergoes transient softening due to initial network weakening by B_12_‐mediated disulfide cleavage, followed by progressive network re‐crosslinking driven by the increased flux of Fc^+^ generated at the anode. As a control, we performed dissipative softening experiments using hydrogels prepared from 8‐arm PEG–SS under the same experimental conditions (2 mM Fc, 50 µM B_12_). Unlike the PR‐based hydrogels, the 8‐arm PEG–SS hydrogels underwent progressive network degradation upon application of the potential, as evidenced by a continuous decrease in the storage modulus (G′) from approximately 2 to 0.3 Pa over 60 min (Figure S23). The films subsequently detached from the electrode owing to extensive network weakening. Increasing the Fc concentration to 8.5 mM results in no significant change in stiffness of polyrotaxane‐based hydrogel over time, as the high flux of Fc^+^ rapidly reoxidizes thiols to disulfides, suppressing their reduction mediated by B_12_^−^ generated at the cathode. As a control, applying −1.0 V in the absence of both redox mediators produced no significant change in the storage modulus (G′) of the hydrogel, confirming that the applied potential alone is insufficient to sustain the dissipative reaction network. The charge consumed during these processes, determined by integration of the chronoamperometric curves (Figure S24), decreases only slightly from −14.5 mC at 0.5 mM Fc to −10.0 mC at 8.5 mM Fc. This modest decrease, despite the increase in Fc concentration, suggests that the fully crosslinked hydrogel partially limits the diffusion of the redox mediators. Nevertheless, the measured charge remains substantially higher than that required for quantitative disulfide bond cleavage (−2.28 mC), calculated according to *Q =* −*2Fn*, where *F* is Faraday's constant and *n* is the number of moles of disulfide bonds. This confirms that a continuous disulfide cleavage and reformation occur during transient softening, which regenerates the crosslinks and prevents network disassembly.

The electrochemical control over hydrogel stiffness under applied potential was further monitored in real time by electrochemical impedance spectroscopy (Figure S25). Measurements performed on hydrogel‐coated electrodes in the parallel‐plate electrochemical cell at −1.0 V over a frequency range of 0.1 MHz to 10 Hz reveal a progressive decrease in interfacial electron‐transfer resistance in the presence of 0.5 mM Fc, consistent with increased permeability of the hydrogel matrix caused by partial network decrosslinking. At 2.0 mM Fc, the initial decrease in resistance is followed by gradual recovery to the original value, whereas at 8.5 mM Fc no significant variation is observed, indicating that the hydrogel network remains unchanged. To confirm that electrochemically induced softening of the hydrogel arises from disulfide decrosslinking, we probe thiol formation using qualitative fluorescein isothiocyanate (FITC) assay [42]. Confocal fluorescence microscopy (Figure S26) shows a marked increase in fluorescence of the hydrogel upon voltage application at low Fc concentration, which subsequently remains constant over time, indicating thiol formation due to predominant B_12_‐mediated disulfide cleavage over Fc‐mediated oxidative recrosslinking. At intermediate Fc concentration, fluorescence intensity increases after about 30 min, corresponding to the minimum in hydrogel stiffness, and subsequently decreases, consistent with Fc^+^‐mediated quantitative reoxidation of thiols to disulfides. At high Fc concentration, no significant fluorescence is detected during voltage application, indicating the absence of thiol formation in the network, which remains in the highly cross‐linked state. The fluorescence intensity profiles obtained from the confocal images (Figure 3d) show that fluorescence increases from the hydrogel–electrode interface and extends into the hydrogel volume, with maximum intensity near the cathode and lower intensity toward the opposite side of the film. Because the confocal images were acquired during electrochemically induced softening, the fluorescence profile reflects the transient spatial distribution of thiols generated during the dynamic remodeling of the hydrogel network. This fluorescence distribution is consistent with the electrochemical mechanism, in which disulfide cleavage and thiol formation are initiated at the cathode by reduced B_12_, while the oxidizing mediator Fc^+^ is generated at the opposite electrode (anode). The spatially heterogeneous thiol distribution therefore indicates that disulfide cleavage does not occur uniformly across the film during electrochemical operation and may result in transient differences in local cross‐link density across the hydrogel thickness. In this context, the changes in G′ measured by rheology reflect the collective mechanical response of the dynamically remodeled polymer network and do not resolve local variations across the hydrogel thickness. These results demonstrate precise temporal modulation of hydrogel stiffness through a fully electrochemical dissipative strategy, in which softening of the polymer network can be selectively sustained or suppressed by controlling the flux of oxidizing and reducing mediators, through the continuous consumption of electrical energy.

### 2.3 Bioelectrochemically Powered Glucose‐Dependent Insulin Release

**FIGURE 4:**
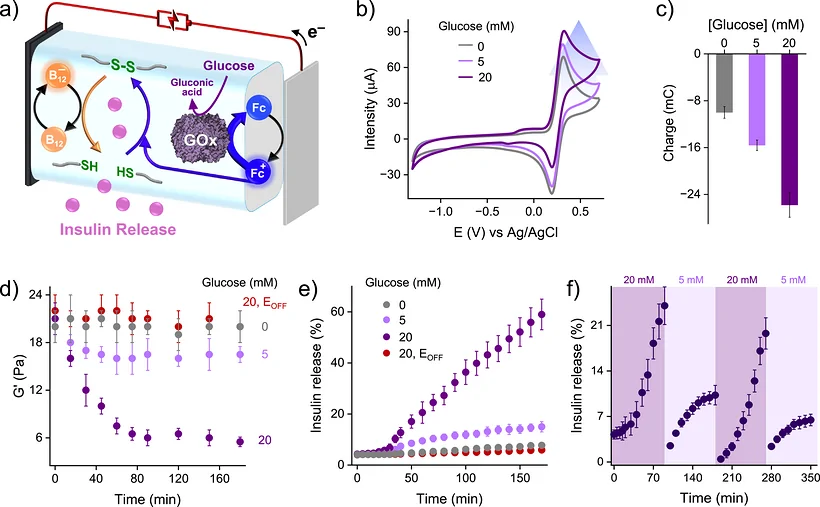
Bioelectrochemically driven glucose‐dependent insulin release. (a) Schematic representation of glucose‐dependent release of FITC‐insulin from a hydrogel loaded with FITC–insulin and GOx. Glucose oxidation provides enzymatic feedback that couples glucose consumption to the antagonistic redox cycle, in which Fc^+^ mediates enzymatic turnover and disulfide recrosslinking within the network. (b) Cyclic voltammograms (10 mV s^−^^1^) recorded under inert atmosphere at working electrode modified with protein‐loaded hydrogel films in a solution containing 50 µM B_12_ and 8.5 mM Fc (0.1 M PBS, pH 7.4) at increasing glucose concentrations (0, 5, and 20 mM). (c) Charge consumed by the antagonistic redox cycle under application of −1.0 V at increasing glucose concentrations. (d) Temporal changes in hydrogel stiffness (*G’*) under application of −1.0 V in a solution containing 50 µM B_12_ and 8.5 mM Fc (PBS 0.1 M, pH 7.4) under inert atmosphere, at increasing glucose concentrations (mM): 0, 5, and 20, and in absence of applied potential (E_OFF_). (e) Time‐dependent release of FITC–insulin from hydrogel‐coated electrodes under application of –1.0 V in a solution containing 50 µM B_12_ and 8.5 mM Fc under inert atmosphere, at increasing glucose concentrations (0, 5, and 20 mM), and in absence of applied potential (E_OFF_). (f) Time‐dependent release of FITC–insulin from hydrogel‐coated electrodes at −1.0 V under inert atmosphere in solution containing 50 µM B_12_ and 8.5 mM Fc, under alternating exposure to solutions containing 20 and 5 mM glucose (90 min in each solution).

Building on this electrochemically sustained platform, we introduce enzymatic feedback regulation into the antagonistic redox cycle to couple the mechanical state of the hydrogel film to metabolic inputs. In particular, oxidoreductase enzymes can utilize redox mediators, including ferrocene derivatives, as artificial electron acceptors during substrate oxidation [43], thereby introducing a competing pathway for Fc^+^ consumption within the mediator cycle. Integration of this enzymatic reaction couples substrate oxidation to the antagonistic redox cycle, allowing the balance between disulfide cleavage and reformation to be dynamically regulated. We therefore demonstrate that this dissipative hydrogel platform can operate as a bioelectrochemically regulated network, when employed in the presence of enzymes, with overall mechanical properties and permeability responding to metabolic signals. Glucose oxidase (GOx) was introduced as a model enzyme that catalyzes glucose oxidation while using Fc^+^ as artificial electron acceptor, thereby providing enzymatic feedback regulation on the mediator balance governing network re‐crosslinking. As a proof‐of‐concept, this out‐of‐equilibrium process enables glucose‐dependent insulin release sustained under continuous electrochemical input (Figure 4a). The development of insulin delivery systems capable of responding to fluctuations in glucose concentration has attracted considerable attention as a promising approach for improving the quality of life of people with diabetes [44, 45]. Glucose‐responsive hydrogels based on glucose oxidase‐mediated pH changes [46, 47, 48], boronic acid–glucose complexation [49, 50, 51], or DNA‐responsive networks [52] have demonstrated proof‐of‐concept glucose‐triggered insulin release. Adaptive insulin delivery systems have been developed to continuously adjust insulin release in response to changing glucose levels through integrated sensing and feedback mechanisms [53, 54]. Electrochemical platforms have also emerged as powerful strategies for integrating real‐time glucose sensing with response‐controlled insulin delivery [55, 56, 57], but they generally rely on device‐level actuation rather than direct regulation of material structure and transport properties. In contrast, our strategy uses glucose not as a direct trigger for insulin release, but to regulate the balance between disulfide cleavage and reformation within the electrochemically powered antagonistic reaction network. Through GOx‐mediated consumption of Fc^+^, glucose dynamically modulates hydrogel permeability and, consequently, insulin release under sustained electrochemical energy input. Unlike conventional stimuli‐responsive hydrogels [34], in which the material passively responds to an external cue, the dissipative reaction network continuously consumes energy to maintain the hydrogel in an out‐of‐equilibrium state. As a result, hydrogel permeability can be reversibly increased and restored over multiple cycles without irreversible network degradation. This coupling of sustained energy input, dynamic network reconfiguration, and enzymatic feedback establishes a bioelectrochemically regulated material platform with adaptive release behavior, representing a step toward future homeostatic insulin delivery systems.

Hydrogel films were electrodeposited in the parallel‐plate electrochemical cell from a solution containing 0.5 mM B_12_, 4 mM Fc, 1 mM 4‐arm PEG‐SH, and 1.5 mM PR–SS, in the presence of 0.1 mg ml^−^^1^ GOx and 0.2 mg ml^−^^1^ FITC‐insulin, by applying −1.0 V for 10 min. The resulting films embed catalytically active GOx (0.083 ± 0.009 U) and a high FITC‐insulin loading (128 ± 9 ng mm^−3^). Evidence for Fc‐mediated glucose oxidation in the GOx‐embedded hydrogel was obtained by CV in a 0.1 M PBS buffer (pH 7.4) containing 50 µM B_12_, 8.5 mM Fc, and increasing glucose concentrations. The 0.1 M PBS buffer provided sufficient buffering capacity to compensate for proton generation during GOx‐catalyzed glucose oxidation. The Fc concentration was maintained at 8.5 mM to ensure efficient mediated electron transfer between GOx and the electrode while keeping the hydrogel in its fully crosslinked state, thereby preventing the antagonistic redox cycle itself from altering the mechanical properties of the hydrogel. As glucose concentration increased, the CVs showed enhanced anodic currents for Fc along with attenuation of the corresponding cathodic peaks (Figure 4b and Figure S27), consistent with Fc^+^‐mediated bioelectrocatalytic oxidation of glucose by GOx. This behavior results in a concomitant increase in the electrochemical charge passed at the electrodes, as determined by integration of chronoamperometric curves recorded during voltage application (Figure 4c). The increase reflects enhanced Fc^+^‐mediated enzymatic turnover at higher glucose concentrations, sustaining the catalytic cycle.

We then investigated the role of enzymatic feedback in modulating the overall mechanical properties of the hydrogel network. An electrode coated with enzyme‐loaded hydrogel films in a parallel‐plate electrochemical cell was subjected to a continuous potential of −1.0 V in the presence of 50 µM B_12_ and 8.5 mM Fc at increasing glucose concentrations under nitrogen, and the stiffness of the hydrogel deposited on the electrode was monitored by rheometry at defined time intervals (Figure 4d). As shown by the spatial distribution of thiols during electrochemically driven hydrogel softening (Figure 3d), local differences in disulfide cleavage can develop over time across the hydrogel thickness, and the measured G′ therefore represents an average value of the integrated mechanical response of the film. In the absence of glucose, no change in hydrogel stiffness is observed (*G’* ≈ 20 Pa), indicating that the network remains fully cross‐linked under the applied potential due to the high flux of Fc^+^ from the anode. The addition of 5 mM glucose (basal concentration) induces only minor changes in stiffness. In contrast, 20 mM glucose (hyperglycemic concentration) results in a decrease in *G’* to ∼6 Pa within ∼75 min, after which the stiffness remains stable. As a control, in the absence of an applied electrochemical potential (E_OFF_), no changes in the hydrogel stiffness are observed under hyperglycemic conditions (G′ ≈ 20 Pa), demonstrating that glucose alone is insufficient to activate the dissipative reaction network. These results are consistent with bioelectrochemical enzymatic feedback regulation of hydrogel stiffness, where electrogenerated Fc^+^ diffusing from the anode serves as the electron acceptor for GOx‐catalyzed glucose oxidation. As the glucose concentration increases, GOx competes more effectively for Fc^+^, decreasing its availability for thiol oxidation and shifting the redox balance toward B_12_‐mediated disulfide cleavage, thereby progressively weakening the hydrogel network.

Having demonstrated glucose‐dependent softening of enzyme‐loaded hydrogels under applied potential, we employed this platform for insulin release. Figure 4e shows the time‐dependent cumulative release profiles of FITC‐insulin from the protein‐loaded hydrogel under voltage application, monitored by fluorescence spectroscopy. The measured cumulative release represents the integrated transport response of the entire hydrogel film. In the absence of glucose, the fully cross‐linked PR‐based hydrogel effectively prevents FITC‐insulin leakage (Figure 4e, gray curve). In the presence of 5 mM glucose, limited insulin release is observed, reaching 15% ± 2% after 170 min. In contrast, 20 mM glucose results in a faster and more pronounced release, reaching 59 ± 6% after 170 min. These results are consistent with enhanced hydrogel decrosslinking at higher glucose concentrations, driven by GOx‐mediated Fc^+^ consumption, which promotes thiol formation and increases the network permeability, enabling diffusion of FITC‐insulin from the weakened hydrogel matrix. Considering the spatially heterogeneous disulfide cleavage discussed above, local changes in cross‐link density and permeability may develop across the hydrogel thickness and influence insulin transport, although these variations are not resolved by the bulk release measurements. The antagonistic redox cycle is therefore essential for achieving glucose‐dependent control over release. In the absence of any applied potential, no insulin release is observed under hyperglycemic conditions, even in the presence of both redox mediators. Notably, the rheological data (Figure 4d) show that hydrogel softening precedes insulin release (Figure 4e), consistent with progressive network remodeling preceding the bulk transport response. Local changes in permeability may therefore precede the bulk release response measured from the entire film. In the absence of Fc, applying voltage with B_12_ alone leads to rapid, nearly quantitative release within 170 min (Figure S28), highlighting the critical role of Fc^+^ in promoting network re‐crosslinking and regulating release kinetics. Crucially, efficient insulin retention and controlled release are observed only in polyrotaxane‐based hydrogel networks. When the 8‐arm PEG–SS control polymer is used instead of PR–SS, the resulting hydrogel films show significantly lower incorporation of both catalytically active GOx (0.053 ± 0.009 U) and FITC‐insulin (30 ± 4 ng mm^−3^). This behavior arises from the lower cross‐link density of the 8‐arm PEG–SS network, which limits protein retention, particularly for the smaller FITC‐insulin. When protein‐loaded 8‐arm PEG–SS films are subjected to voltage in the absence of glucose, FITC‐insulin is released spontaneously, and increasing glucose concentration does not significantly affect either the release kinetics or the total amount released (Figure S29). These results demonstrate that incorporation of polyrotaxane into the hydrogel network yields a highly cross‐linked matrix that suppresses spontaneous leakage in the fully cross‐linked state and enables precise stiffness modulation under dissipative conditions, thereby allowing glucose‐controlled insulin release.

To evaluate the responsiveness of the platform under fluctuating glucose levels, the hydrogel‐coated electrode was alternately transferred every 90 min into fresh release media containing either high (20 mM) or low (5 mM) glucose concentrations (Figure 4f). Under continuous voltage application, exposure to 20 mM glucose resulted in rapid FITC‐insulin release, reaching a cumulative release of 24% ± 3% with a corresponding release rate of 0.24% min^−1^, calculated as the first‐order derivative of the release profile. Upon transfer to fresh insulin‐free medium containing 5 mM glucose, the release rate decreased to 0.04% min^−1^, with only an additional 10% ± 5% insulin released over the following 90 min. Re‐exposure to 20 mM glucose restored rapid release, yielding a further 20% ± 5% release and increasing the release rate to 0.26% min^−1^. A second transfer to 5 mM glucose again suppressed release, resulting in only an additional 6 ± 1% insulin released and a release rate of 0.04% min^−1^. Overall, cumulative FITC‐insulin release reached 60% after 360 min of cyclic glucose exposure.

The reduced release at low glucose concentrations is consistent with diminished enzymatic consumption of Fc^+^, allowing Fc^+^‐mediated disulfide reformation to dominate and restore a less permeable network. These results demonstrate the reversibility of the process and the adaptive behavior of the dissipative reaction network, which dynamically adjusts hydrogel permeability in response to changes in glucose concentration. Consequently, insulin release is promoted under hyperglycemic conditions and suppressed as glucose levels decrease. Overall, this bioelectrochemically sustained dissipative hydrogel integrates continuous electrochemical energy input with enzymatic feedback to dynamically regulate network structure, mechanics, and permeability, thereby enabling adaptive glucose‐dependent insulin release.

## 3 Conclusion

In conclusion, we have established a fully electrochemical dissipative platform for independently controlling the growth and operation of disulfide‐crosslinked hydrogel films. By electrochemically generating reduced vitamin B_12_ at the cathode and oxidized ferrocene at the anode, we achieve precise control over disulfide cleavage and reformation without the need for sacrificial chemical reagents, thereby programming the crosslink density, composition, and lifetime of the hydrogel through the applied potential and the duration of electrochemical energy input. This approach advances electrofabrication beyond equilibrium assembly by introducing a voltage‐driven antagonistic redox cycle that controls network crosslinking and decrosslinking, enabling transient, out‐of‐equilibrium modulation of network structure and properties. The polyrotaxane‐based hydrogel exhibits greater robustness than the non‐mechanically interlocked control hydrogel during redox‐driven network remodeling, highlighting the potential of mechanically interlocked architectures for more durable dissipative materials.

We further demonstrate that this dissipative hydrogel platform can operate as a bioelectrochemically regulated membrane, in which mechanics and permeability are governed by enzymatic feedback that operates through competition for ferricenium within the antagonistic redox cycle. As a proof‐of‐concept, GOx‐loaded hydrogels enabled glucose‐dependent insulin delivery under continuous electrochemical energy input, with release enhanced under hyperglycemic conditions and suppressed as glucose levels decreased. The ability to dynamically regulate permeability in response to fluctuating glucose levels distinguishes this platform from conventional glucose‐responsive materials, which typically rely on passive swelling or one‐time triggering. More broadly, this work establishes a general strategy for coupling electrochemically sustained dissipative reaction networks to the regulation of mechanics and molecular transport in soft materials through biochemical feedback. Extension of this strategy to other oxidoreductase enzymes provides a versatile route to programmable hydrogel films for biointerfaces, controlled delivery, and adaptive soft devices powered by electrical energy and regulated by diverse biochemical signals.

## 4 Experimental Section

### 4.1 Materials

All reagents were purchased from commercial suppliers and used without further purification. 4‐arm thiol‐terminated polyethylene glycol (4‐arm PEG‐SH, MW 5000) and 8‐arm thiol‐terminated polyethylene glycol (tripentaerythritol core) (8‐arm PEG‐SH, MW 10000) were purchased from Jenkem Technology USA (Plano, TX). Tris(2‐carboxyethyl)phosphine hydrochloride (TCEP, >98%), 2,2’ dipyridyl disulfide and 2‐aminoethanethiol hydrochloride were purchased from TCI Europe N.V. All other reagents were purchased from Sigma Aldrich Co (St. Louis, MO).

### 4.2 Preparation of Hydrogel Films

Hydrogel films were electrodeposited using a parallel‐plate electrochemical cell with a 2 mm interelectrode distance. A solution containing 4.0 mM ferrocenecarboxylic acid (Fc, 1.9 mg) and 0.5 mM vitamin B_12_ (B_12_, 1.4 mg) was prepared in phosphate‐buffered saline (PBS, 2.0 ml) 0.1 M at pH 7.4 under an inert atmosphere, in the presence of 1 mM 4‐arm thiol terminated polyethylene glycol (4‐arm PEG‐SH, MW 5000, 10 mg) and 1.5 mM disulfide‐functionalized polyrotaxane (PR‐SS, 62 mg; see Supporting Information for synthesis and characterization). For electrodeposition of the hydrogel film, 100 µl of this solution were transferred to the electrochemical cell, and a constant potential (−0.8, −1.0, and −1.2 V) was applied for variable durations (2, 5, 10, 20, 30, and 60 min). For the electrodeposition of the control hydrogel films based on 8‐arm disulfide functionalized PEG (8‐arm PEG‐SS), the solution of 4.0 mM Fc, 0.5 mM B_12_ and 1 mM 4‐arm PEG‐SH was prepared in 2.0 ml of PBS 0.1 M at pH 7.4 under an inert atmosphere, in the presence of 1.3 mM 8‐arm PEG‐SS (28 mg). This solution (100 µl) was transferred in the electrochemical cell and the deposition of the film was carried out by applying −0.8 V for 20 min, −1.0 V for 10 min, and −1.2 V for 5 min. After electrodeposition, the films were thoroughly washed with deionized water to remove unreacted species and subsequently stored in deionized water.

### 4.3 Preparation of Protein‐Loaded Hydrogel Films

Hydrogel films loaded with glucose oxidase (GOx, from *Aspergillus Niger*, 152 U/mg) and fluorescein isothiocyanate‐labelled (FITC)‐insulin were prepared following the procedure described in *Paragraph 4.2*, with slight modifications. A 0.2 mg/ml FITC‐insulin solution was prepared in PBS 0.1 M (pH 7.4), in the presence of 4 mM Fc, 0.5 mM B_12_, 1 mM 4‐arm PEG‐SH, and either 1.5 mM PR‐SS or 1.3 mM 8‐arm‐PEG‐SS. The resulting mixtures (100 µl) was combined with 2 µl of a 5.0 mg/ml GOx solution in PBS 0.1 M (pH 7.4), then transferred to the electrochemical cell under inert atmosphere. Hydrogel films were deposited by applying a constant potential of −1.0 V for 10 min. The resulting hydrogel films were thoroughly washed with deionized water and subsequently immersed in deionized water for 2 h to remove weakly adsorbed proteins.

## Author Contributions

**Roberto Baretta**: conceptualization, investigation, writing – original draft, methodology, validation, formal analysis, data curation. **Marco Frasconi**: conceptualization, writing – review and editing, supervision, funding acquisition, methodology.

## Conflicts of Interest

The authors declare no conflicts of interest.

## Supporting information




**Supporting File**: advs77934‐sup‐0001‐SuppMat.pdf.

## Data Availability

The data that support the findings of this study are available from the corresponding author upon reasonable request.
