# Supplementary material for: Bioelectrochemically Powered Out‐of‐Equilibrium Hydrogels Enable Feedback‐Regulated Functions
Source: Adv Sci (Weinh). 2026 Sep 27:e77934. Online ahead of print. doi: 10.1002/advs.77934 (PMC13616324; doi:10.1002/advs.77934)
Supplement: Supplementary file 1 [file ADVS-9999-e77934-s001.pdf]

Supporting Information

Bioelectrochemically Powered Out-of-Equilibrium Hydrogels Enable Feedback-Regulated Functions

Roberto Baretta,[a] and Marco Frasconi*[a]

[a]Department of Chemical Sciences, University of Padova, Via Marzolo 1, 35131 Padova, Italy

*E-mail: marco.frasconi@unipd.it

Table of Contents

1. Instrumentation……………………………………………………………………………S2

2. Supporting Methods…………………………………………………………………………S3

3. NMR Characterization……………………………………………………………………S12

4. Characterization of Hydrogel Films……………………………………………………S18

4.1 Electrochemical Characterization of the Mediated Electrochemical Cycle

4.2 Determination of Electron Transfer Rate Constant

4.3 Fully Electrochemical Transient Hydrogel Growth

4.4 Determination of Hydrogel Films Composition

4.5 Electrodeposition of Hydrogel Films Under Equilibrium Conditions

4.6 Electrodeposition of Control Hydrogel Films (8-Arm PEG-SS)

4.7 Rheological Characterization of Hydrogel Films

5. Fully Electrochemical Transient Hydrogel Softening…………………………………S33

6. Bioelectrochemically Controlled Insulin Release………………………………………S36

6.1 (Bio)-Electrochemical Characterization

6.2 Release of FITC-Insulin from Protein-Loaded Hydrogels

7. References………………………………………………………………………………S39

1. Instrumentation

Ultraviolet–visible (UV–vis) spectra were collected using an Agilent Cary 60 spectrophotometer. All electrochemical measurements were recorded on potentiostat/galvanostat PGSTAT204 (Metrohm Autolab B.V.). A parallel-plate electrochemical cell with 2 mm interelectrode distance, with an Ag/AgCl (3M KCl) as reference electrode, a platinum slide as counter electrode (anode) and glassy carbon disk (diameter (Ø): 4 mm) as working electrode (cathode) was used for the electrochemical deposition and characterization of the hydrogel films. All the electrochemical potentials are reported versus Ag/AgCl (3M KCl) reference electrode. Scanning electron microscopy (SEM) analysis was performed at CEASC (Centro di Analisi e Servizi per la Certificazione, University of Padova, Italy), employing a Quanta 200 Environmental SEM (FEI Company, Hillsboro, OR) with a thermionic emission gun and an Xplore 30 energy dispersive X-ray (EDX) detector (Oxford Instruments, Abingdon, UK). Images were collected in low vacuum mode (0.53 torr), with an accelerating voltage of 20 kV. Rheological characterizations were performed on Kinexus Lab+ rheometer (Malvern Instruments, UK) with a 20 mm plate geometry at a fixed gap. Frequency sweeps were performed from 0.01 to 100 Hz at 1 % strain. Strain sweeps were performed from 0.1 to 1000 % strain at 1 Hz. Nuclear magnetic resonance (NMR) experiments were performed using a Bruker 400 MHz Avance III HD, operating at 400.13 MHz for ^{1}H experiments and at 100.61 MHz for ^{13}C experiments, equipped with a BBI-z grad 5 mm probe head. The NMR spectra were processed using Topspin 3.5 software and the chemical shift scale was calibrated using the residual solvent signal. Confocal laser microscopy was performed on a Zeiss LSM 900 with Airyscan 2 equipped with Zeiss EC Plan–Neofluar 10x/0.5 objective employing an excitation wavelength of 488 nm. The images were processed using the Zeiss ZEN and ImageJ software. Fluorescence spectra were collected on FLS1000 fluorimeter by Edinburgh Instruments, using a quartz cuvette of 1 cm path length.

2. Supporting Methods

2.1) Synthesis of Disulfide Linker

The following synthesis was performed according to a reported procedure with minor modifications.[1]

2.1.1 Synthesis of 2-(Pyridyldithio)-Ethylamine Hydrochloride (PDA–HCl)

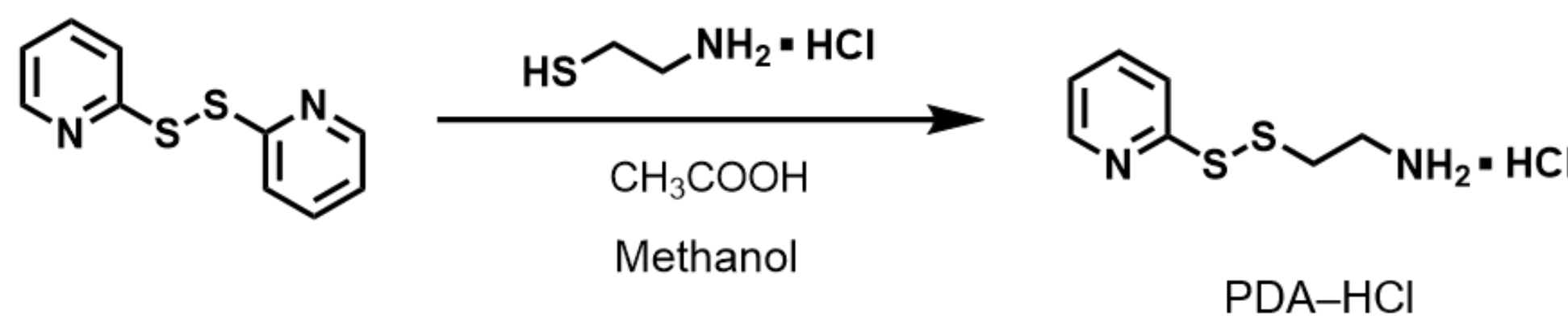

Scheme S1. Synthesis of PDA–HCl.

Under an argon (Ar) atmosphere, 2-aminoethanethiol hydrochloride (1.29 g, 11.3 mmol, 1 eq) was dissolved in 10 ml of methanol. This solution was added dropwise under vigorous magnetic stirring to 24 ml solution of methanol containing 0.91 ml of glacial acetic acid and 2,2'-dipyridyl disulfide (5.0 g, 22.7 mmol, 2 eq). The resulting yellow mixture was stirred at room temperature for 48 h in the dark under an Ar atmosphere, followed by concentration in vacuo, providing a yellow viscous liquid. The resulting product was precipitated by addition of cold diethyl ether (60 ml), collected by centrifugation (6000 rpm, 2 min), and the supernatant discarded. The solid was purified by six cycles of dissolution in methanol (8 ml), followed by reprecipitation with cold diethyl ether (60 ml), centrifugation, and removal of the supernatant. The product was then dried in vacuo to obtain 2-(pyridyldithio)-ethylamine hydrochloride (PDA-HCl) (1.76 g, 70% yield) as a white solid. ^{1}H NMR (400 MHz, D_2O, 298 K) δ: 8.47 (d, J = 4.80 Hz, 1H), 7.86 (t, J = 7.64 Hz, 1H), 7.77 (m, 1H), 7.35 (t, J = 6.18 Hz, 1H), 3.35 (t, J = 6.26 Hz, 2H), 3.12 (t, J = 6.26 Hz, 2H). ^{13}C NMR (100 MHz, DMSO-d_6, 298 K) δ: 158.11, 149.81, 137.91, 121.60, 120.01, 37.66, 34.73.

2.1.2 Synthesis of 2-(Pyridyldithio)-Ethylamine (PDA)

Under an Ar atmosphere, sodium hydroxide (73 mg, 1.8 mmol) was dissolved in 373 µl of degassed DI water and the resulting solution was added to 227 µl of a degassed DI water solution, containing 2-(pyridyldithio)-ethylamine hydrochloride (364 mg, 1.6 mmol). The resulting mixture was vigorously stirred and then allowed to stand for 30 min to enable phase separation. The transparent lower organic phase was collected and isolated as the PDA free base. ^{1}H NMR (400 MHz, $CDCl_3$, 298 K) δ: 8.48 (d, J = 4.52 Hz, 1H), 7.71-7.59 (m, 2H), 7.13-7.06 (m, 1H), 3.00 (t, J = 6.14 Hz, 2H), 2.88 (t, J = 6.04 Hz, 2H).

2.2) Synthesis of Polyrotaxane

The following synthesis was performed according to a reported procedure with minor modifications.[2]

2.2.1 Synthesis of PEG(COOH)$_2$

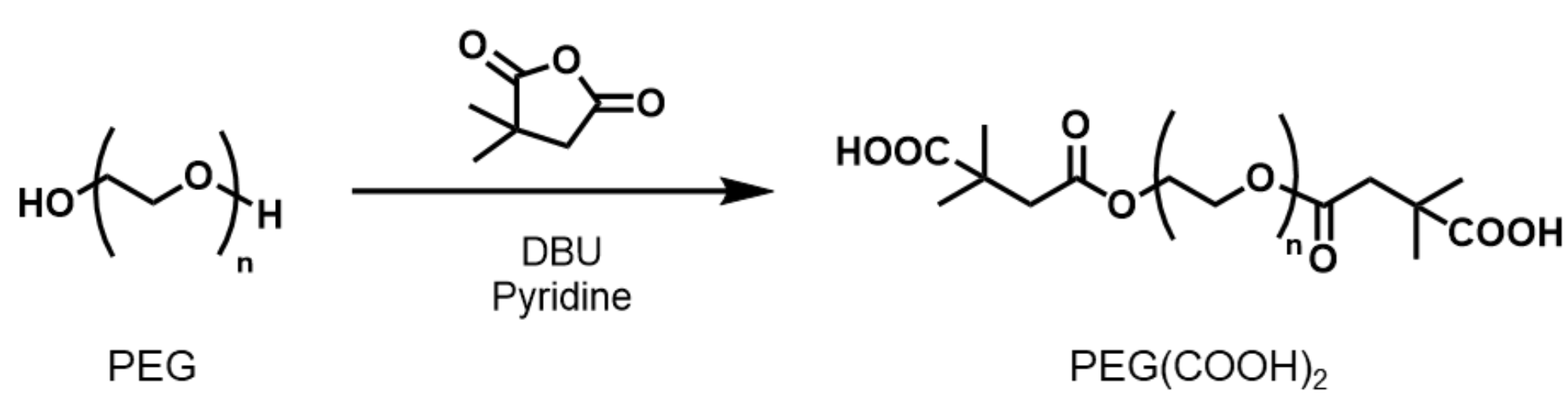

Scheme S2. Synthesis of PEG(COOH)$_2$.

Under an Ar atmosphere, polyethylene glycol (PEG, MW 10000, 5.94 g, 0.59 mmol, 1 eq) was dissolved into 30 ml of anhydrous pyridine, followed by the addition of 1,8-diazabicyclo[5.4.0]undec-7-ene (DBU, 148 µl, 0.97 mmol, 1.6 eq) and 2,2-dimethylsuccinic

anhydride (98%, 4.0 g, 31.0 mmol, 52 eq). The reaction mixture was stirred at 60 °C overnight under an Ar atmosphere. The reaction mixture was subsequently added dropwise to deionized water (125 ml), and the pH was adjusted to 2 by addition of concentrated hydrochloric acid (37%). The product was extracted with dichloromethane, and the combined organic phases were dried over anhydrous sodium sulfate, filtered, and concentrated under reduced pressure to afford PEG(COOH)$_2$ (5.32 g, 88% yield) as a white solid. ^{1}H NMR (400 MHz, DMSO-d_6, 298 K) δ: 12.20 (s, 2H); 4.62-3.38 (m, 1070H); 1.16 (12H). ^{13}C NMR (100 MHz, DMSO-d_6, 298 K) δ: 177.70, 172.25, 69.77, 63.30, 43.52, 25.04.

2.2.2 Synthesis of Polyrotaxane (PR)

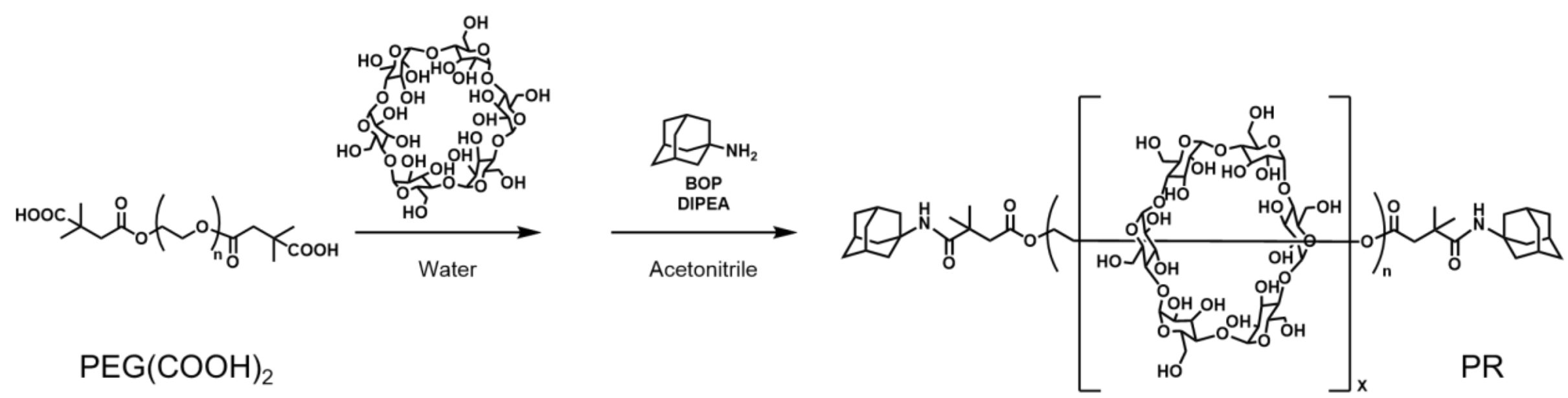

Scheme S3. Synthesis of polyrotaxane (PR).

PEG(COOH)$_2$ (1.00 g, 98 μmol, 1 eq) was dissolved in DI water (7 ml), and combined with a saturated aqueous solution of α-cyclodextrin (α-CD, 4.0 g, 4.0 mmol, 41 eq, in 30 ml of DI water). The resulting mixture was stirred at room temperature for 1 h, and then stored at 4 °C for 3 days, leading to the formation of a white precipitate. The mixture was freeze-dried to afford a white powder. The obtained solid was dispersed in anhydrous acetonitrile (40 ml) under an Ar atmosphere. A solution of 1-adamantylamine (134 mg, 0.86 mmol, 8.8 eq), benzotriazole-1-yl-oxy-tris-(dimethylamino)-phosphonium hexafluorophosphate (BOP, 392 mg, 0.86 mmol, 8.8 eq) and N-ethyldiisopropylamine (DIPEA, 152 μl, 0.88 mmol, 9.0 eq) in anhydrous acetonitrile (10 ml)

was then added. The resulting suspension was stirred at room temperature for 48 h under Ar. The mixture was centrifuged (5000 rpm, 7 min), and the collected solid was washed twice with acetonitrile and dried under vacuum. The product was then dissolved in dimethyl sulfoxide (DMSO, 33 ml) and added dropwise to DI water (330 ml) under stirring. The resulting solution was purified by dialysis (1 week), followed by freeze-drying to afford the polyrotaxane (1.55 g, 82% yield) as a white solid. ^{1}H NMR (400 MHz, DMSO-d_6, 298 K) δ: 5.63 (d, 56H; J = 6.32 Hz); 5.45 (s, 60H); 4.84 (s, 58H); 4.45 (s, 57H); 3.96-3.338 (m, 1215 H); 2.02 (s; 6H); 1.98-1.88 (m, 10H); 1.64 (s, 9H); 1.33-1.11 (m, 17H).

2.3) Functionalization of Polyrotaxane

2.3.1 Synthesis of Disulfide-Functionalized Polyrotaxane (PR–SS)

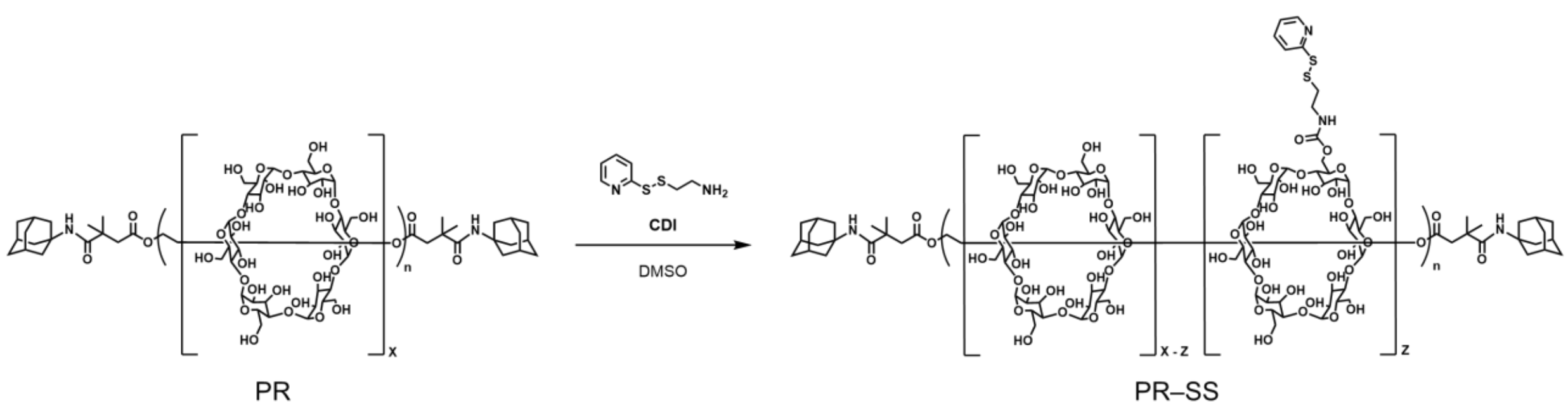

Scheme S4. Synthesis of disulfide-functionalized polyrotaxane (PR–SS).

CD–PR (200 mg, 10.4 µmol, 1 eq) was dissolved in anhydrous DMSO (4 ml) under an Ar atmosphere. 1,1'-carbonyldiimidazole (185 mg, 1.11 mmol, 107 eq) was added, and the reaction mixture was stirred for 30 min at room temperature. Subsequently, 2-(pyridyldithio)ethylamine (249.6 mg, 1.34 mmol, 129 eq) was added, and the mixture was stirred overnight at room temperature under Ar. The reaction mixture was then added dropwise to diethyl ether (80 ml), resulting in precipitation of the product. The solid was collected and washed sequentially with

dichloromethane and diethyl ether, and dried under reduced pressure to afford PR–SS (190 mg, 88% yield) as a white solid. ^{1}H NMR (400 MHz, DMSO-d_6, 298 K) δ: 8.50 (s, 7H); 7.93-7.77 (m, 15H); 6.18-3.44 (m, 1520 H); 2.02 (s; 6H); 1.98-1.86 (m, 10H); 1.64 (s, 10H); 1.36-1.05 (m, 17H).

2.4) Synthesis of Disulfide-Functionalized 8-arm PEG (8-arm PEG–SS)

The following synthesis was performed according to a reported procedure with minor modifications.[3]

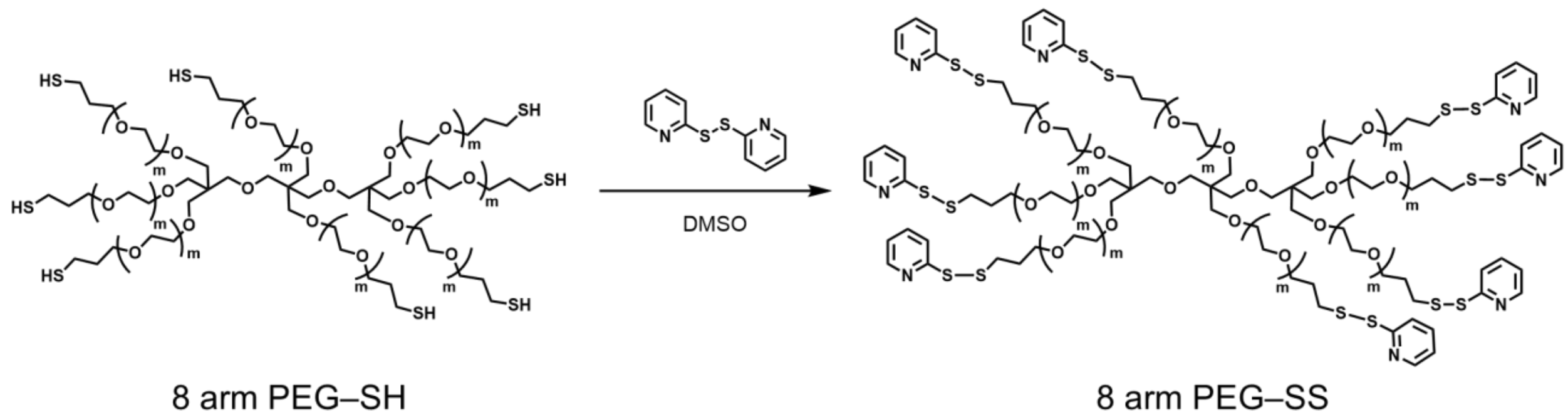

Scheme S5. Synthesis of disulfide-functionalized 8-arm PEG (8-arm PEG-SS).

8-arm thiol terminated PEG (8-arm PEG-SH, MW 10000, tripentaerythritol core; 104 mg, 10.4 μmol, 1 eq) was dissolved in anhydrous DMSO (3 ml) under an Ar atmosphere. 2,2'-dipyridyl disulfide (145 mg, 0.65 mmol, 63 eq) was added and the reaction mixture was stirred overnight at room temperature under Ar in the dark. The reaction mixture was then added dropwise to diethyl ether (70 ml), resulting in precipitation of the product. The solid was collected, washed three times with diethyl ether, and dried under reduced pressure to obtain 8-arm PEG-SS (101 mg, 89% yield) as a white solid. ^{1}H NMR (400 MHz, DMSO-d_6, 298 K) δ: 8.48 (d, J = 4.52 Hz, 8H); 8.04-7.82 (m, 15H); 7.32-7.24 (m, 8H); 4.15-3.38 (m, 1070 H). ^{13}C NMR (100 MHz, DMSO-d_6, 298 K) δ: 159.52, 149.38, 137.80, 121.06, 119.11, 69.76.

2.5) Thiol Quantification in the Hydrogel Films

The thiol content of the electrodeposited films was quantified using a reported spectrophotometric method[6] following hydrogel dissolution. Hydrogel-coated electrodes were immersed in 600 µl of a 60 µM TCEP solution in 0.1 M PBS (pH 7.4) and stirred for 1 h to ensure complete film dissolution. After removal of the electrode, 5 µl of a 50 mM 4-chloro-7-nitrobenzofurazan (NBD-Cl) solution in DMSO were added, and the resulting mixture stirred for an additional 30 min. UV–vis spectra were recorded using a quartz cuvette (1 cm path length), and the absorbance at 423 nm was used to determine the thiol concentration based on a calibration curve obtained from 4-arm PEG-SH solutions with known thiol concentrations (10–80 µM). The equation of the calibration curve for thiols is: $y = -0.039 + 0.0059\ \mu M^{-1}\ [\text{–SH}]$

2.6) Determination of PR:4-arm PEG Ratio by ^{1}H NMR Spectroscopy

The PR:4-arm PEG ratio in the electrodeposited hydrogel films was determined by ^{1}H NMR spectroscopy following film dissolution. Hydrogel-coated electrodes were immersed in 600 µl of 0.1 M PBS (pH 7.4) prepared in D_2O, in the presence of 60 µM TCEP, and the mixture was stirred for 2 h to ensure complete dissolution of the film. After removal of the electrode, the solution was transferred to an NMR tube, and the ^{1}H NMR spectrum was recorded.

2.7) Rheological Characterization

Hydrogel-coated electrodes, prepared according to our approach (see *Experimental Section Paragraph 4.2*), were used as the working electrode in a parallel-plate electrochemical cell (2 mm

inter-electrode distance). Under an inert atmosphere, a PBS solution (0.1 M, pH 7.4) containing B_{12} (50 μM) and different concentrations of Fc (0.5, 2.0, and 8.5 mM) was added in the electrochemical cell, and a constant potential of -1.0 V was applied. Following removal of the working electrode from the electrochemical cell at defined time intervals, the mechanical properties of the hydrogels were evaluated using a rheometer equipped with a temperature-controlled parallel-plate configuration. Rheological measurements were performed in fixed-gap mode using the hydrogel-coated glassy carbon electrode directly as the lower plate of the rheometer and a stainless-steel parallel plate as the upper geometry. After completion of the electrochemical experiment, the hydrogel-coated electrode was removed from the electrochemical cell and directly transferred to the rheometer without detaching the hydrogel from the electrode. Measurements were performed at a frequency of 1 Hz and a strain of 1%, at a constant temperature of 25 °C.

2.8) Characterization of Hydrogel Films by Confocal Laser Microscopy

The formation of thiols during the electrochemical-driven softening of the hydrogel films was evaluated by confocal laser microscopy. Hydrogel-coated electrodes were subjected to a constant potential of -1.0 V in 0.1 M PBS (pH 7.4) containing B_{12} (50 μM) and varying concentrations of Fc (0.5, 2.0, and 8.5 mM) under inert atmosphere. At defined time intervals, the hydrogel-coated electrodes were removed, rinsed with water, and immersed in a 30 μM fluorescein isothiocyanate (FITC) solution in PBS for 30 min. The hydrogels were then soaked in degassed DI water for 1.5 h to remove unreacted FITC. Confocal images were acquired using an excitation wavelength of 488 nm along the z-axis perpendicular to the hydrogel surface. The fluorescence intensity of each frame was extracted, and the average fluorescence intensity across all frames was calculated.

2.9) Glucose Oxidase Activity Assay

The activity of GOx embedded within the hydrogel was quantified using a 2,2′-azino-bis(3-ethylbenzothiazolinesulfonic acid) diammonium salt (ABTS)/horseradish peroxidase (HRP, 200 U mg^{-1}) coupled assay, following a previously reported procedure with minor modifications.[7] Hydrogel-coated electrodes were immersed in 1.5 ml of 0.1 M PBS (pH 7.4) containing HRP (6 U), ABTS (2.4 mM), and glucose (10 mM). After 60 s of incubation, the UV–vis spectrum of the solution was recorded to determine enzyme activity. One unit of activity is defined as the amount of GOx that catalyzes the formation of 1 μmol of product per minute under the specified conditions.

2.10) Rheological Characterization of Bioelectrochemically-Driven Hydrogel Softening

Electrodes coated with protein-loaded hydrogels, prepared according to our approach (see *Experimental Section Paragraph 4.3*), were used as the working electrode in a parallel-plate electrochemical cell (2 mm interelectrode distance), with a platinum slide serving as the counter electrode. Under an inert atmosphere, a 0.1 M PBS solution (pH 7.4) containing 50 μM B_{12} and 8.5 mM Fc, in the presence of different concentrations of glucose (0, 5, and 20 mM), was introduced and allowed to equilibrate for 30 min prior to applying a constant potential of -1.0 V. Following the removal of the working electrode from the electrochemical cell at defined time intervals, the mechanical properties of the hydrogels were evaluated using a rheometer equipped with a temperature-controlled parallel-plate geometry. Measurements were performed in fixed-gap mode using stainless steel probe and the hydrogel-coated glassy carbon electrode as base, at a frequency of 1 Hz and a strain of 1%, at a constant temperature of 25 °C.

2.11) Insulin Release Study

To monitor FITC-insulin release, protein-loaded hydrogel-coated electrodes were used as the working electrode in a parallel-plate electrochemical cell, with a platinum slide as the counter electrode. The electrochemical cell was immersed in 4 ml of 0.1 M PBS solution (pH 7.4),

comprising 50 µM B_{12} and 8.5 mM Fc in the presence of different concentrations of glucose (0 mM, 5 mM and 20 mM), under an inert atmosphere. A constant potential of -1.0 V was applied throughout the experiment. FITC-insulin release was monitored by fluorescence spectroscopy (λ_{ex} = 490 nm; λ_{em} = 520 nm). At defined time intervals, 10 µl aliquots of the solution were collected, diluted 100-fold in 0.1 M PBS (pH 7.4), and analyzed. The concentration of released FITC-insulin was determined using a calibration curve generated from FITC-insulin standard solutions (0.01–1 ng ml^{-1}) in 0.1 M PBS (pH 7.4). The total amount of FITC-insulin loaded in the hydrogel films was determined by complete dissolution of the network upon addition of 60 µM TCEP in 0.1 M PBS (pH 7.4). The release percentage was calculated by normalizing the amount of FITC-insulin released under each condition to the total amount of FITC-insulin loaded in the hydrogel.

For release experiments under alternating glucose conditions, protein-loaded hydrogel-coated electrodes were subjected to a constant potential of -1.0 V under an inert atmosphere for 90 min in 4 ml of 0.1 M PBS (pH 7.4) containing 50 µM B_{12}, 8.5 mM Fc, and 20 mM glucose. The electrode was then transferred to 4 ml of 0.1 M PBS (pH 7.4) containing 50 µM B_{12}, 8.5 mM Fc, and 5 mM glucose and maintained under the same conditions for an additional 90 min. This cycle was repeated on the same hydrogel sample by alternately exposing it to solutions containing 20 mM and 5 mM glucose. At each transfer, the release medium was completely replaced with fresh solution, and the amount of insulin released was quantified independently for each 90 min interval.

3. NMR Characterization

3.1 PDA–HCl

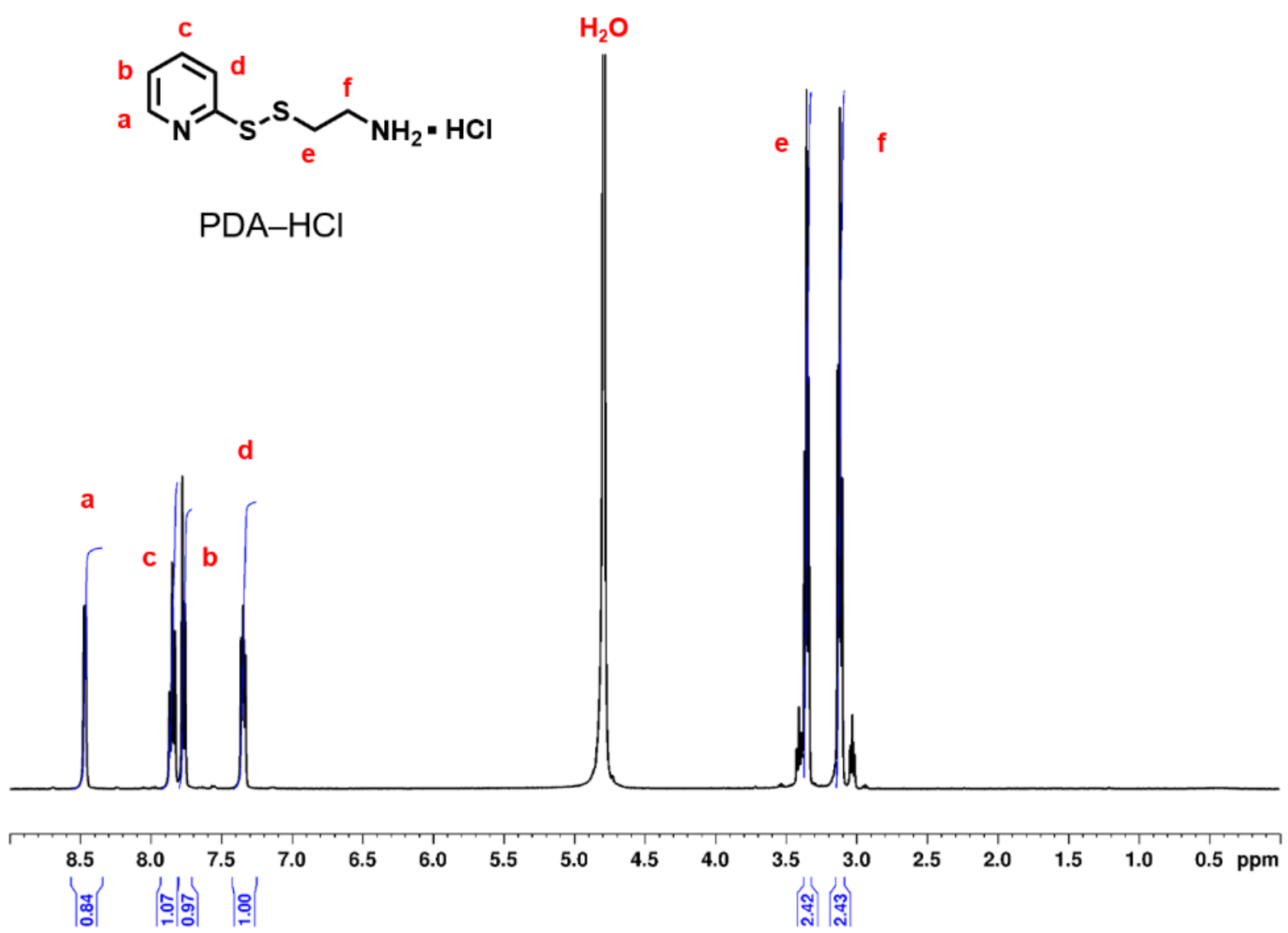

Figure S1. ^{1}H NMR spectrum (400 MHz, D_2O, 298 K) of PDA–HCl.

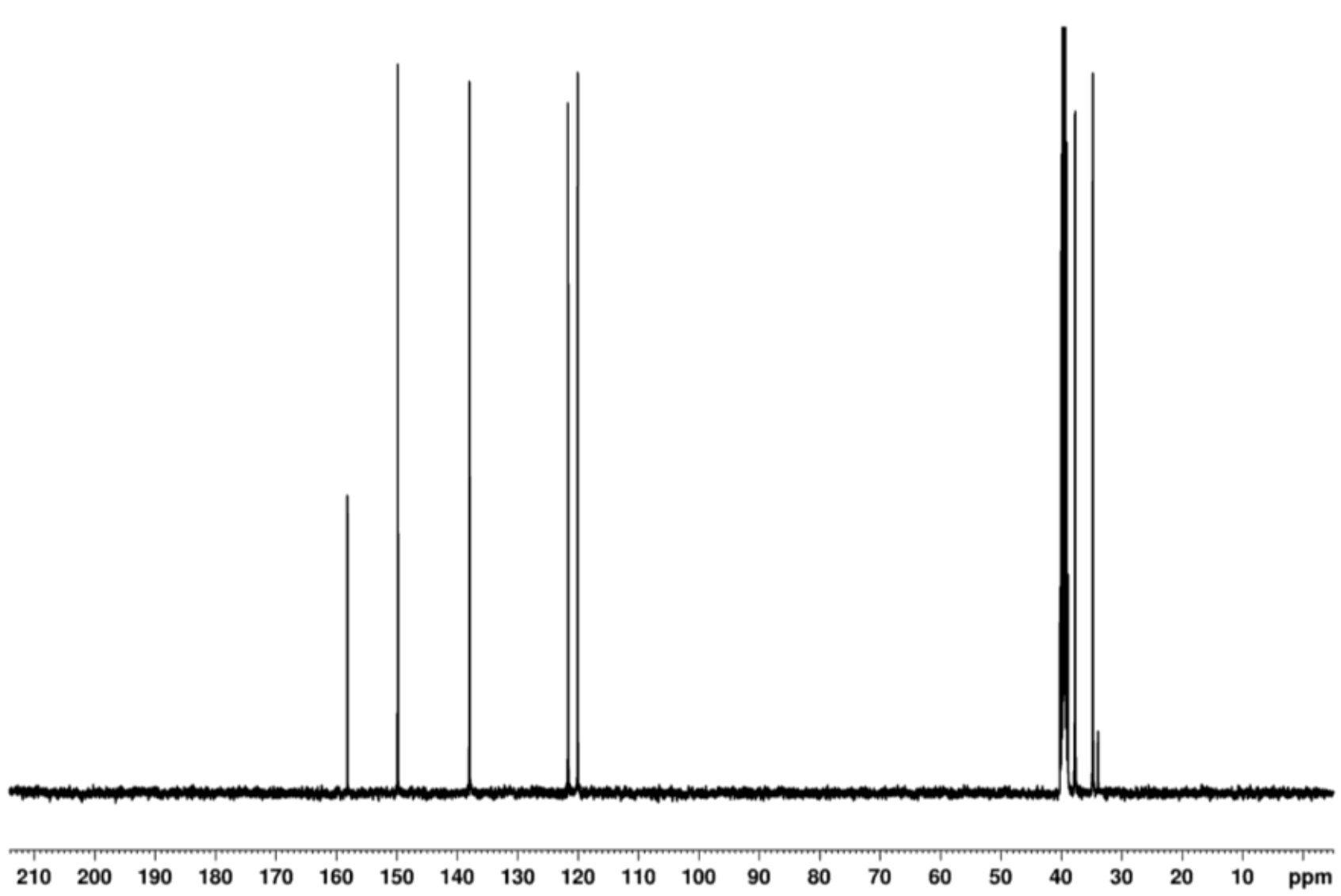

Figure S2. ^{13}C NMR spectrum (100 MHz, DMSO-d_6, 298 K) of PDA–HCl.

3.2 PDA

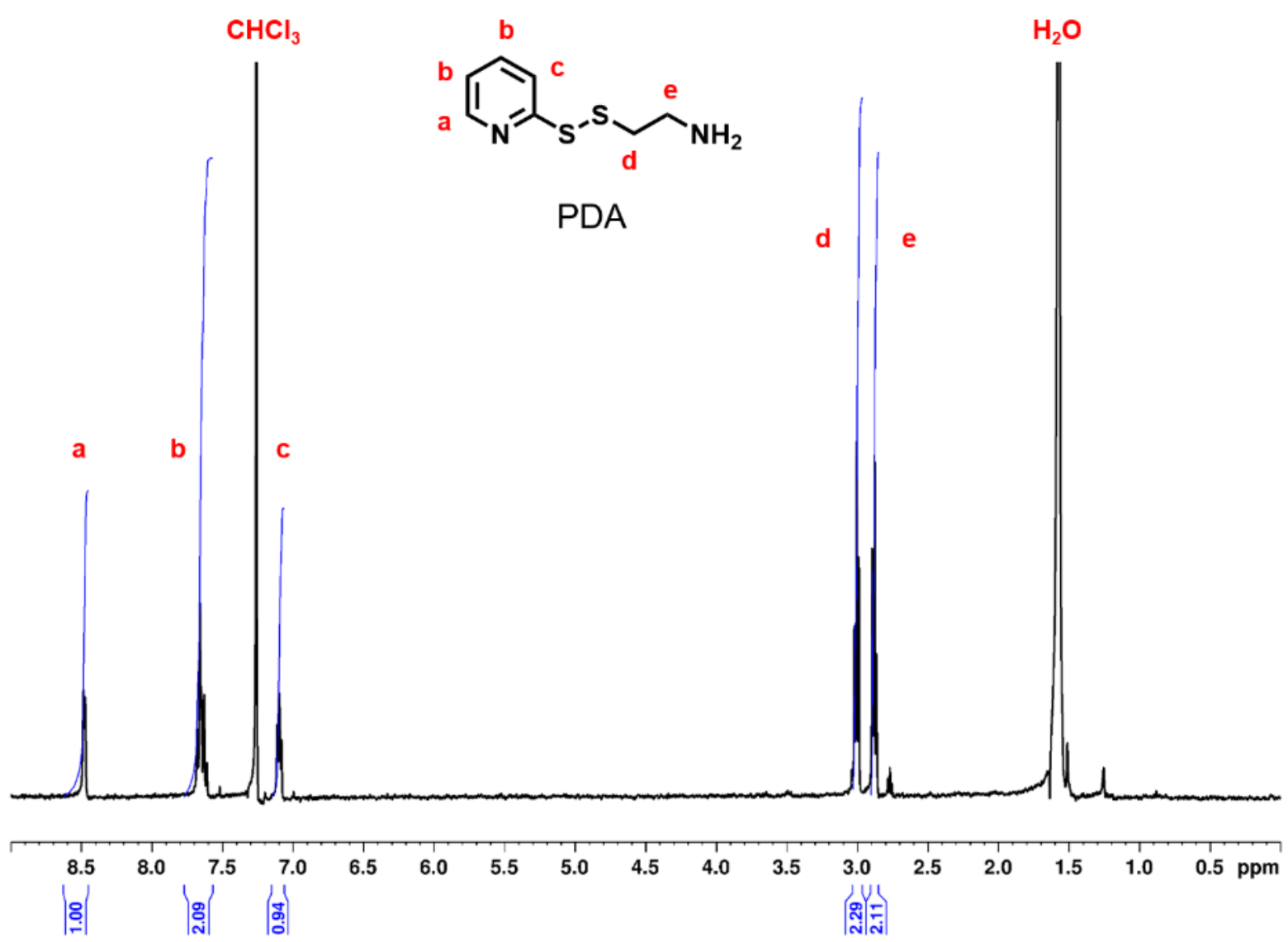

Figure S3. ^{1}H NMR spectrum (400 MHz, $CDCl_3$, 298 K) of PDA.

3.3 PEG(COOH)₂

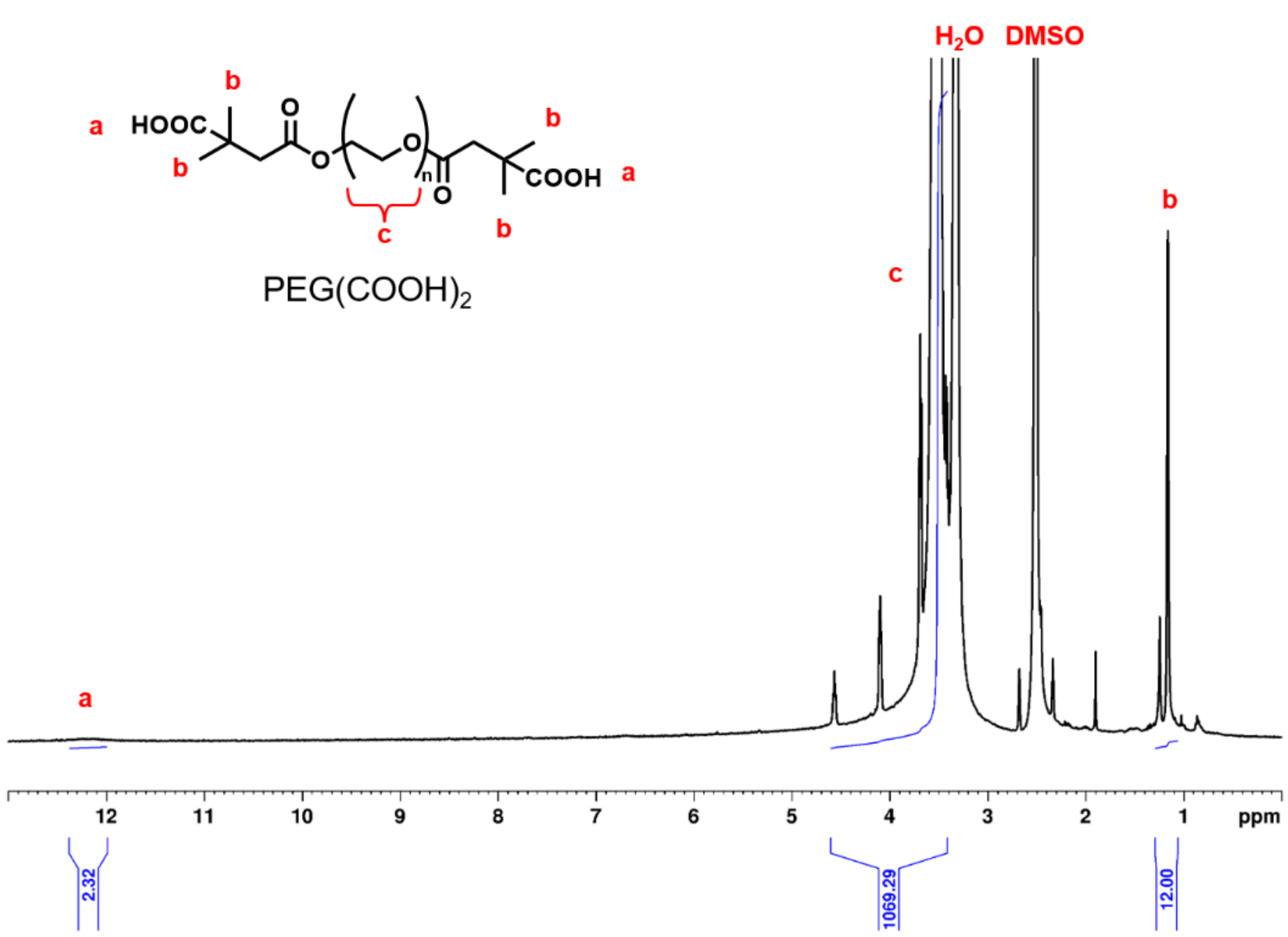

Figure S4. ^{1}H NMR spectrum (400 MHz, DMSO-d_6, 298 K) of $PEG(COOH)_2$.

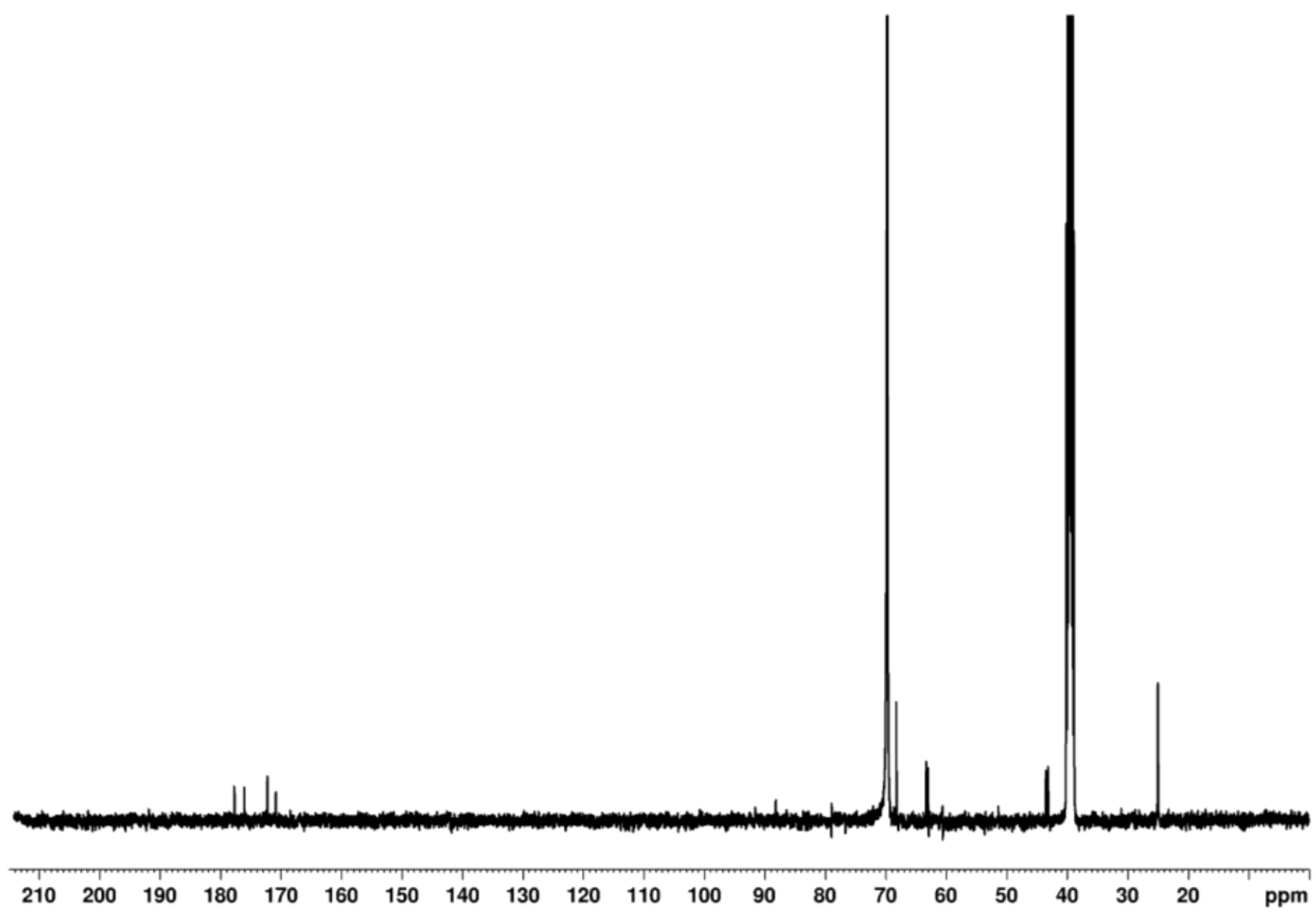

Figure S5. ^{13}C NMR spectrum (100 MHz, DMSO-d_6, 298 K) of $PEG(COOH)_2$.

3.4 Polyrotaxane (PR)

The formation of polyrotaxane, obtained by threading α-cyclodextrin rings (α-CDs) through a PEG chain, was confirmed by ^1H NMR spectroscopy (Figure S6), and found to be consistent with previously reported results.[2,4] The spectrum shows signals corresponding to the adamantane end groups capping the $PEG(COOH)_2$ dumbbell, along with characteristic shifts in the α-CD proton resonances compared to those of unthreaded α-CDs.[5] These features confirm the successful threading of α-CD rings along the PEG axle and the formation of the polyrotaxane structure. The average number of α-CD units threaded per PEG chain was estimated from the integral of the α-CD proton signal (**f**), normalized by the six equivalent protons per α-CD, yielding approximately 9 α-CD units per polyrotaxane.

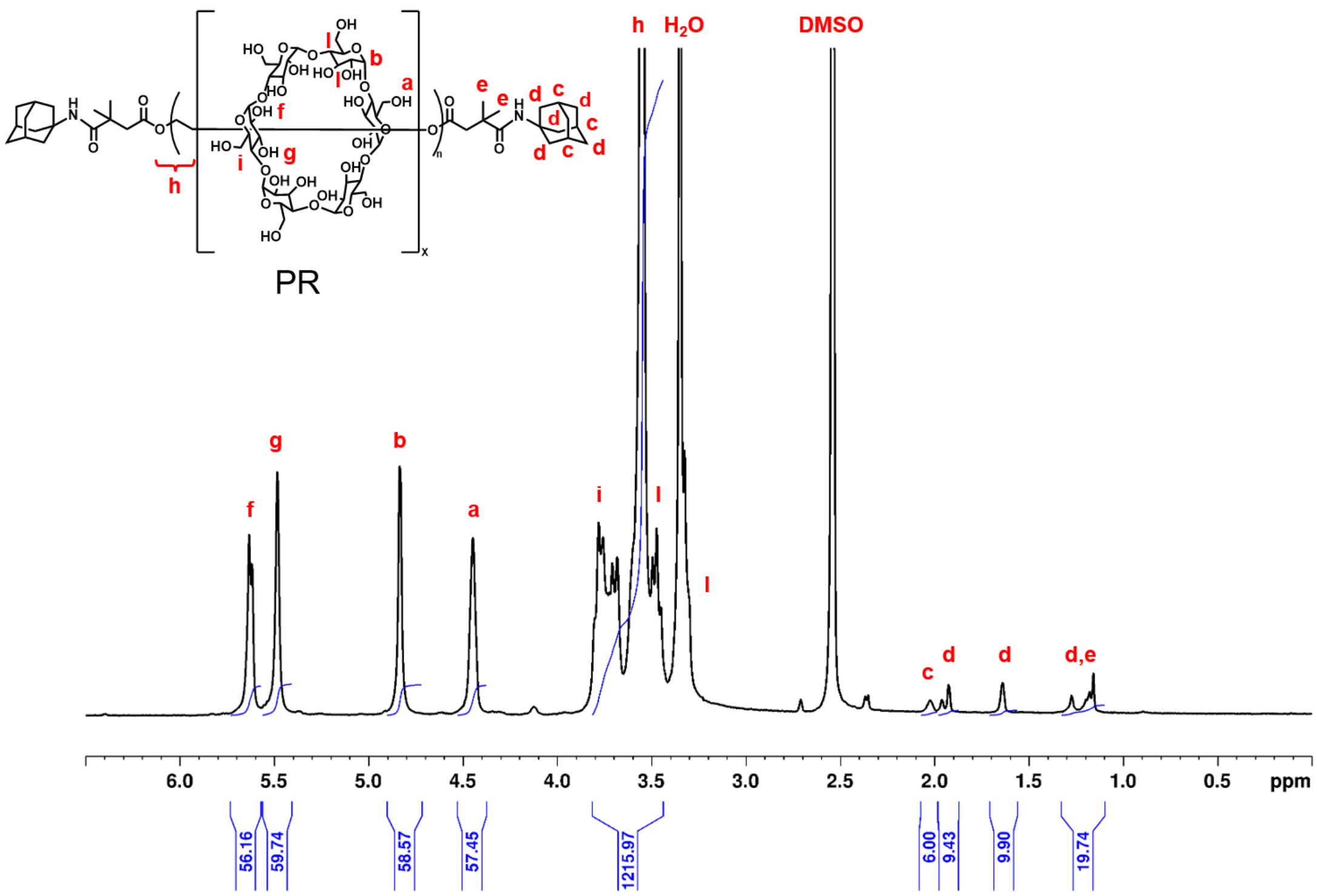

Figure S6. ^{1}H NMR spectrum (400 MHz, DMSO-d_6, 298 K) of polyrotaxane (PR).

3.5 Disulfide Functionalized Polyrotaxane (PR–SS)

The functionalization of the polyrotaxane (PR) with 2-(pyridyldithio)ethylamine (PDA) side chains was confirmed by ^{1}H NMR spectroscopy (Figure S7). The spectrum shows characteristic signals of the pyridyl units (**a**, **b**), along with noticeable changes in the shape and chemical shift of the resonances assigned to α-CD protons (**g**), consistent with covalent modification of the threaded cyclodextrins. The average number of PDA groups per polyrotaxane was estimated by integrating the proton signal of the pyridyl unit (**a**), using the adamantane –CH protons (**c**) as an internal reference (set to 6). This analysis yields an average of approximately seven PDA substituents per polyrotaxane.

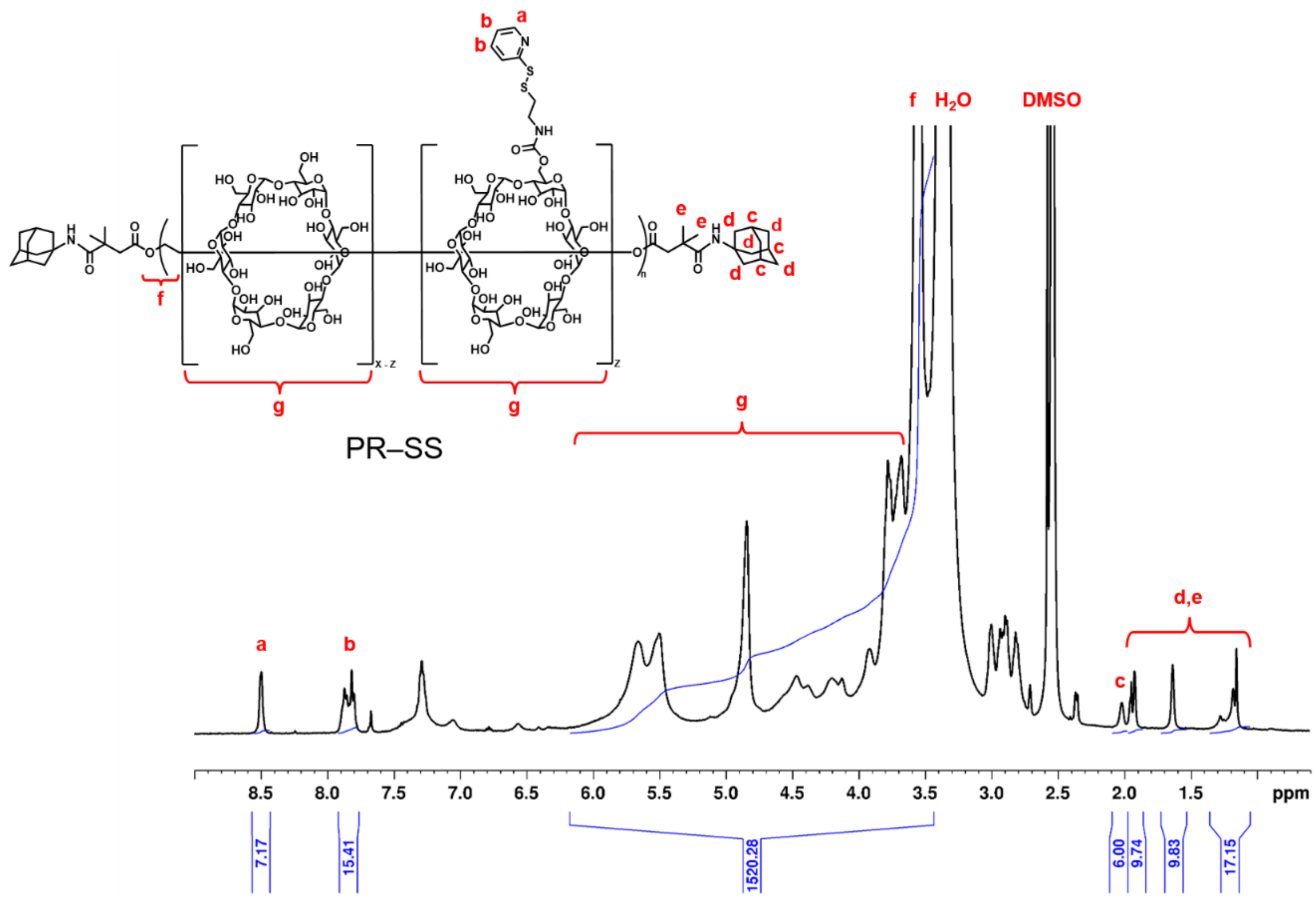

Figure S7. ^{1}H NMR spectrum (400 MHz, DMSO-d$_6$, 298 K) of disulfide-functionalized polyrotaxane (PR–SS).

3.6 Disulfide-Functionalized 8-arm PEG (8-arm PEG-SS)

The functionalization of thiols groups of 8-arm PEG-SH with mercaptopyridine was confirmed by ^{1}H NMR spectroscopy (Figure S8). The spectrum shows characteristic signals of the pyridyl units (**a**, **b** and **c**), along with the signals corresponding to the PEG chain (**d**). The average number of mercaptopyridine groups per 8-arm-PEG molecule was estimated by integrating the proton signal of the pyridyl unit (**a**), using the PEG protons (**d**) as an internal reference (set to 1070, corresponding to the expected value for PEG with MW 10000). This analysis yields an average of 8 mercaptopyridine groups per 8-arm PEG, indicating a complete functionalization of the –SH groups of the polymer.

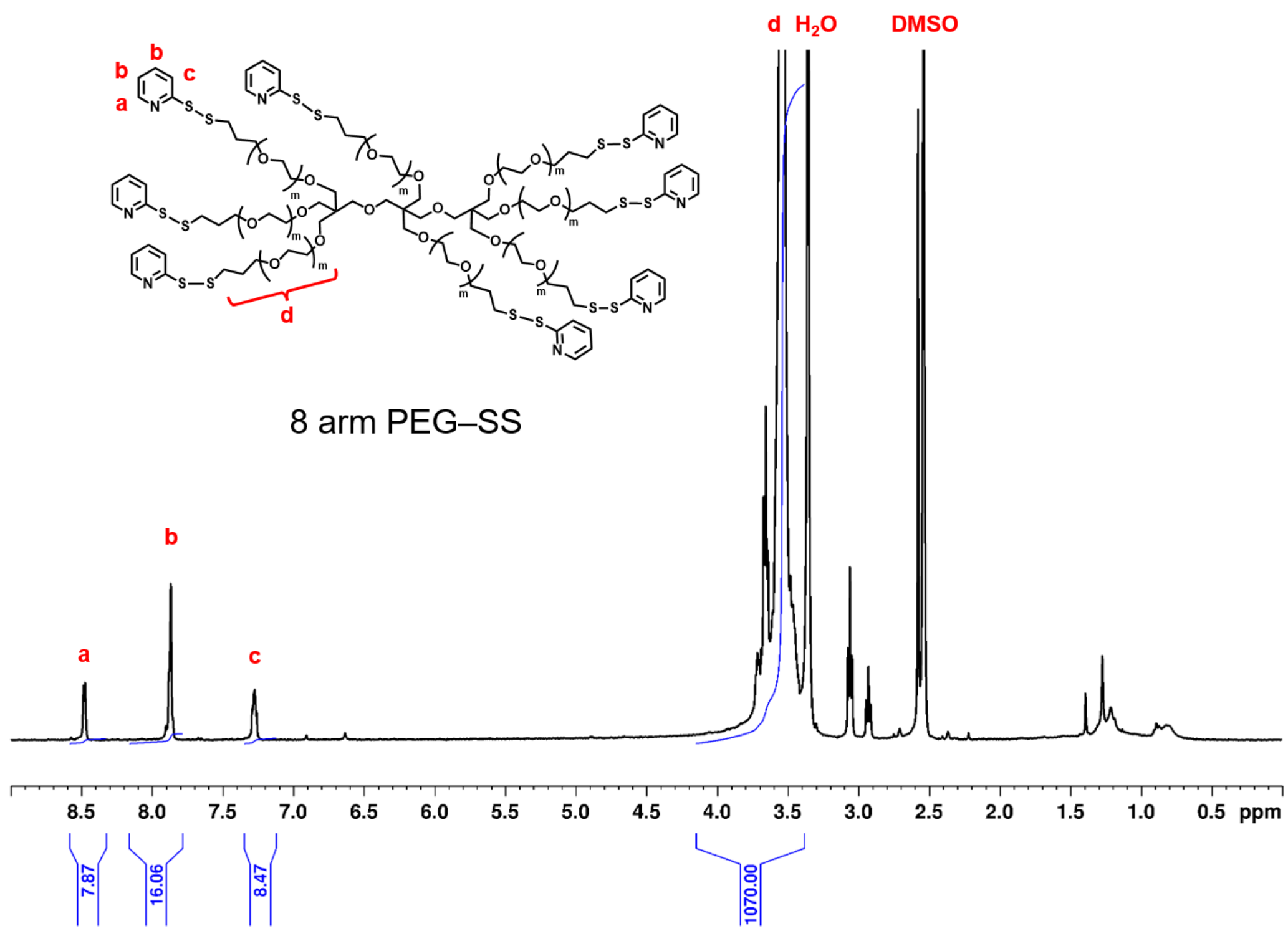

Figure S8. ^{1}H NMR spectrum (400 MHz, DMSO-d_6, 298 K) of disulfide-functionalized 8-arm PEG (8-arm PEG-SS).

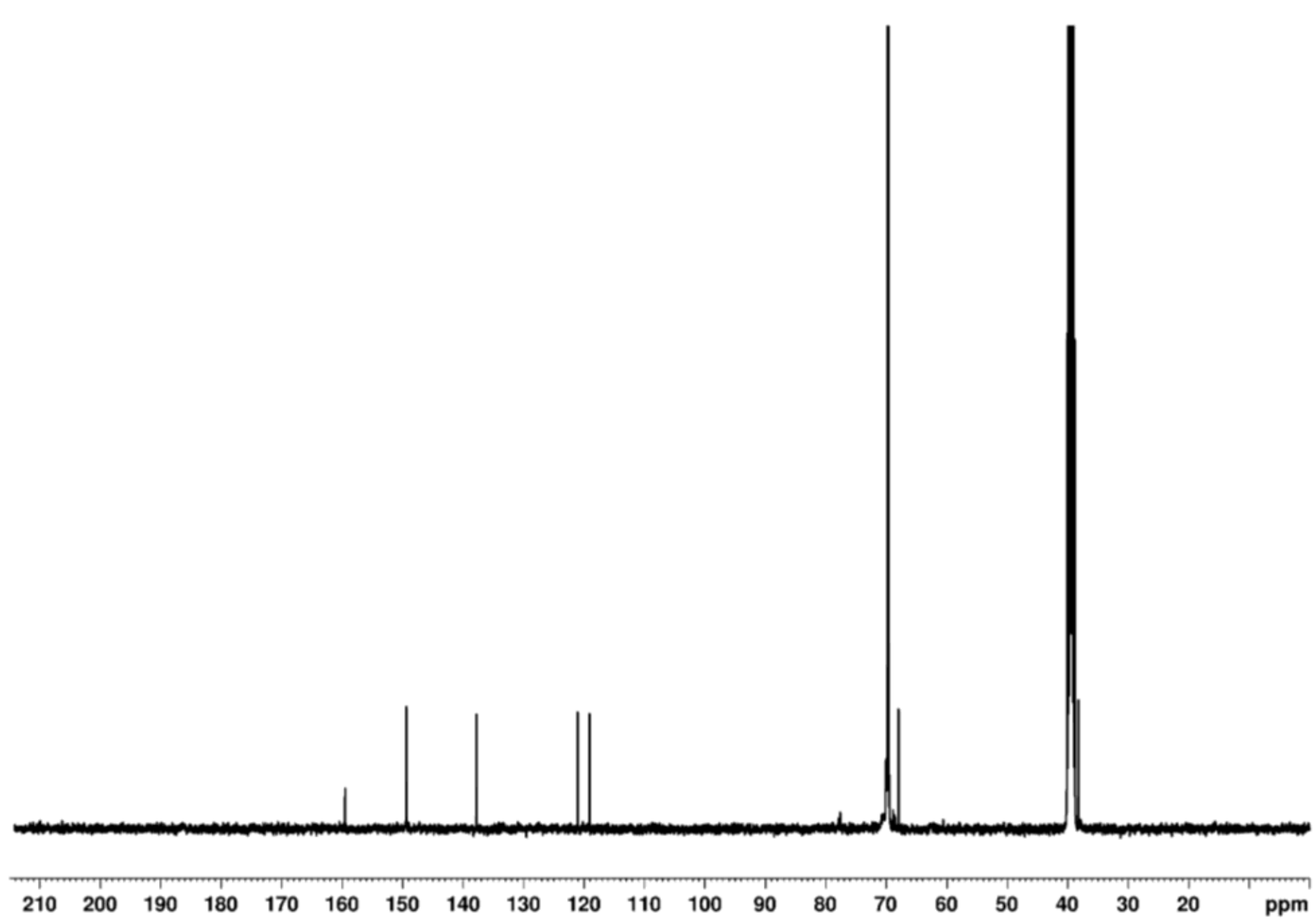

Figure S9. ^{13}C NMR spectrum (100 MHz, DMSO-d_6, 298 K) of 8-arm PEG-SS.

4. Characterization of Hydrogel Films

4.1) Electrochemical Characterization of the Mediated Electrochemical Cycle

Cyclic voltammetry (CV) was performed under inert atmosphere in PBS solutions (0.1 M, pH 7.4) containing 100 µM B_{12} alone, 50 µM Fc alone, and a mixture of both mediators (Figure S10). The peak potentials and current intensities observed for the individual mediators are preserved in the mixed system, indicating reversible behavior and the absence of electrochemical interference between the two mediators when subjected to the application of the electrochemical potential.

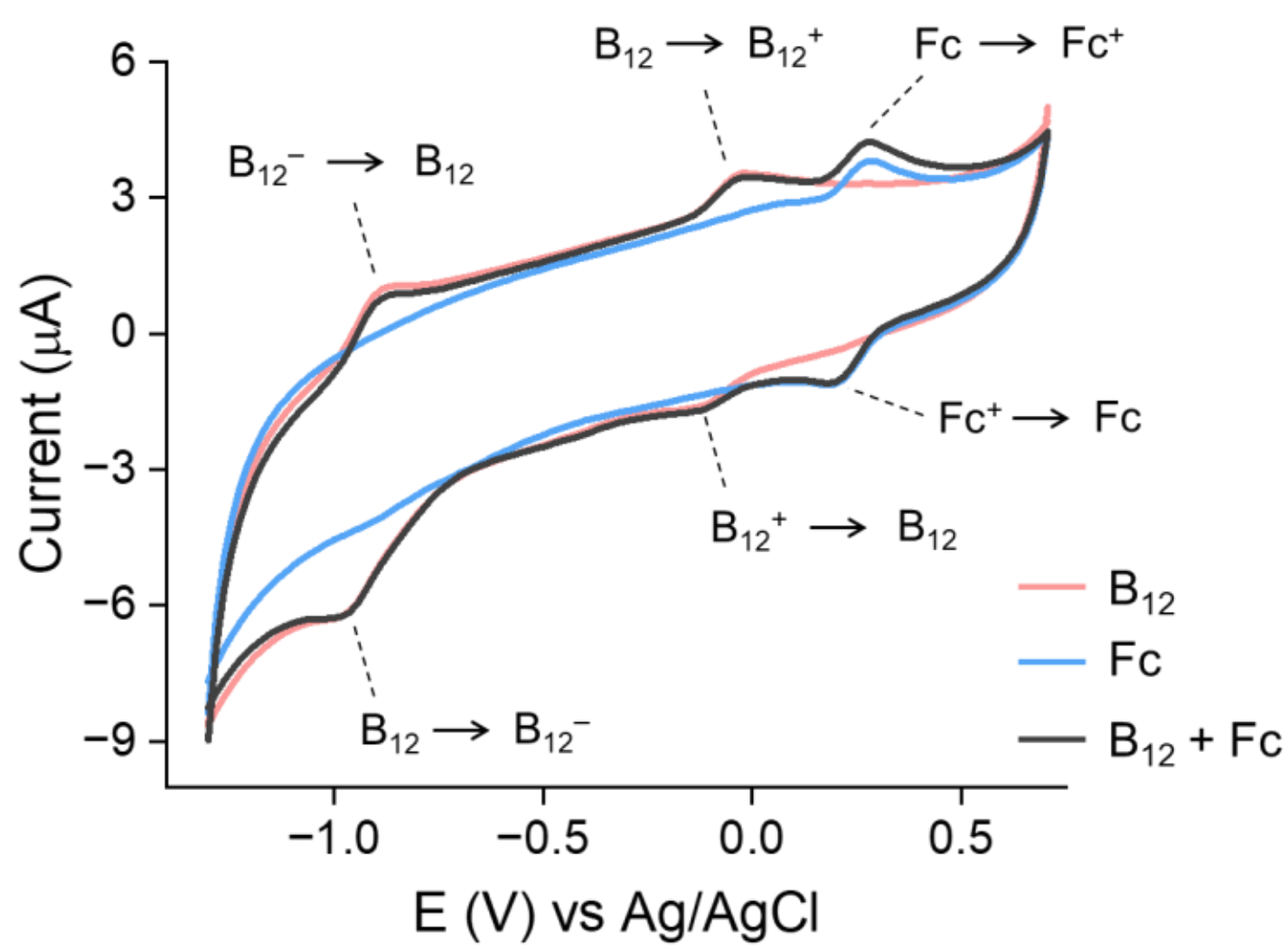

Figure S10. Cyclic voltammograms (250 mV/s) of 50 µM Fc (blue curve) and 100 µM B_{12} (pink curve) solution alone and solution of both mediators (black curve) in 0.1 M PBS (pH 7.4).

To verify that PR–SS must first be reduced by vitamin B_{12}^- generated at the electrode before being oxidized by Fc^+, cyclic voltammetry was performed under inert atmosphere by initiating the scan from 0 V toward positive potentials (Figure S11).

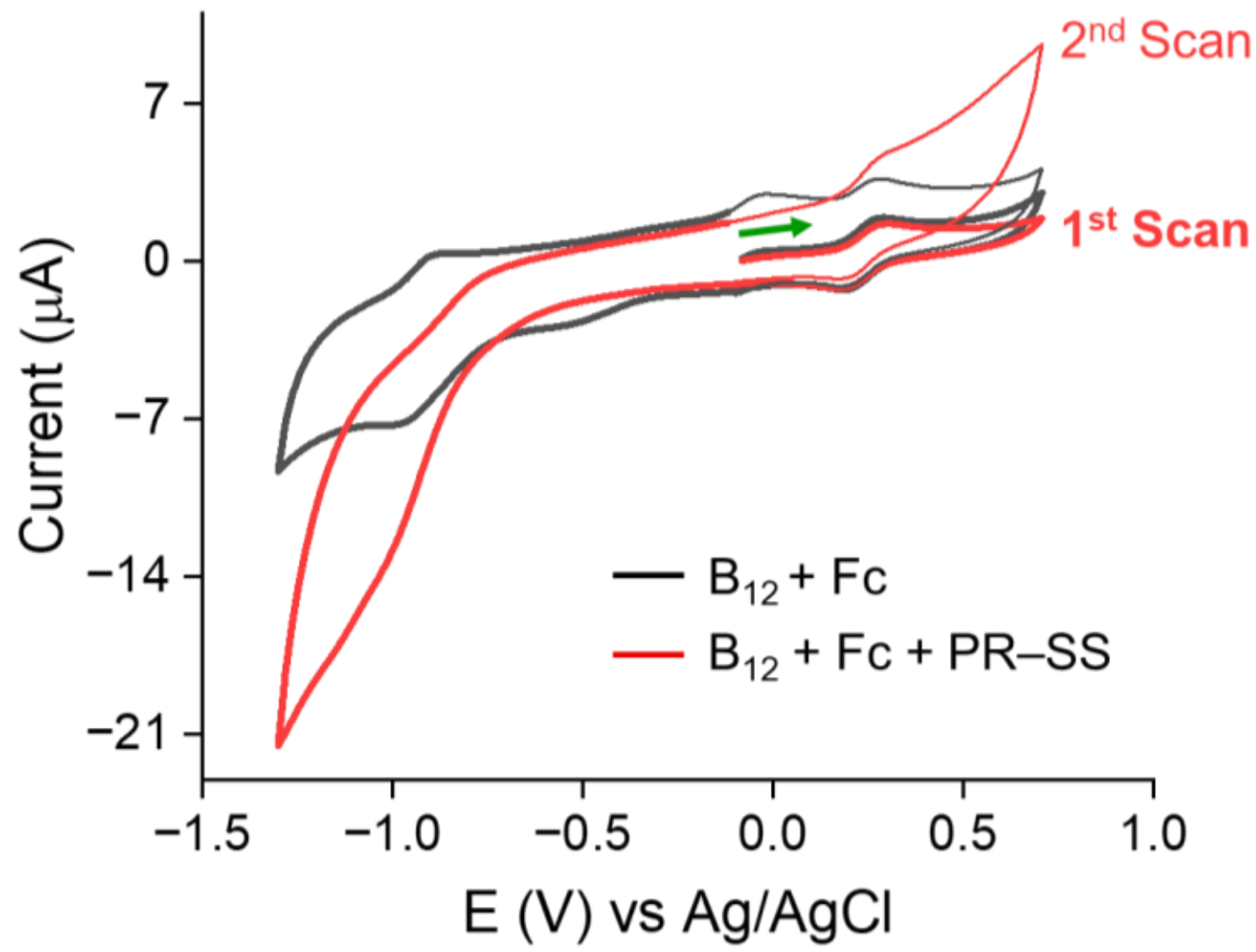

Figure S11. Cyclic voltammograms (250 mV s^{-1}) recorded in 0.1 M PBS (pH 7.4) for a solution containing 50 µM Fc and 100 µM B_{12} in the absence (black curves) and presence (red curves) of 50 µM PR–SS. The potential was scanned from 0 V toward positive values (first scan: bold line; second scan: thin line).

The first scan shows the characteristic oxidation peak of Fc (red curve) with an intensity comparable to that observed for a solution of Fc in the absence of PR–SS (black curve), followed by a cathodic catalytic current associated with the B_{12}-mediated reduction of disulfide to thiols. In the subsequent scan, a catalytic anodic current for Fc is observed, arising from the Fc-mediated oxidation of the thiols, generated in the previous reductive step, back to disulfides.

4.2) Determination of Electron Transfer Rate Constant

The homogeneous electron transfer rate constant (k_{et}) for the reductive redox-cycling mechanism of vitamin B_{12} (B_{12}) in the presence of disulfide-functionalized polyrotaxane (PR–SS) was determined by cyclic voltammetry, according to the theory developed for the E_rC_i’ catalytic mechanism.[8] In this model, an electrochemically reversible step (E_r) is coupled to a chemically irreversible reaction (C_i'). Specifically, the B_{12}/B_{12}^{-} redox couple undergoes reversible electron

transfer at the electrode surface, while the reduced form B_{12}^{-} participates in an irreversible one-electron reduction of disulfide groups in the PR–SS substrate in solution.

Under pure kinetic conditions, where substrate consumption is negligible, the voltammogram of the mediator in the presence of the substrate exhibits a characteristic sigmoidal (S-shaped) profile between the forward and reverse scans. As shown in Figure S12, such S-shaped voltammograms were obtained at a glassy carbon disk electrode (Ø = 3 mm) over a range of scan rates (50–500 mV s^{-1}) in 0.1 M PBS (pH 7.4), using 100 µM B_{12} as the mediator and 50 µM PR–SS as the substrate. Under those conditions, the E_rC_i' mechanism can be described by the following equation:

$$\frac{i_c}{i_p} = \frac{1}{0.4463}\sqrt{\left(\frac{RT}{nFv}\right)k_{obs}}$$

where i_c is the plateau catalytic current of the mediator in the presence of the substrate, i_p is the peak current of the mediator in the absence of the substrate, F = 96485 C mol^{-1}, R = 8.314 J mol^{-1} K^{-1}, and T = 294.15 K, n = 1 (number of electrons), and v the potential scan rate expressed in V s^{-1}. From the experimentally determined k_{obs}, the homogeneous electron transfer rate constant can be calculated according to:

$$k_{obs} = k_{et}C_A^0$$

where C_A^0 = 350 µM is the disulfide concentration of the PR–SS substrate.

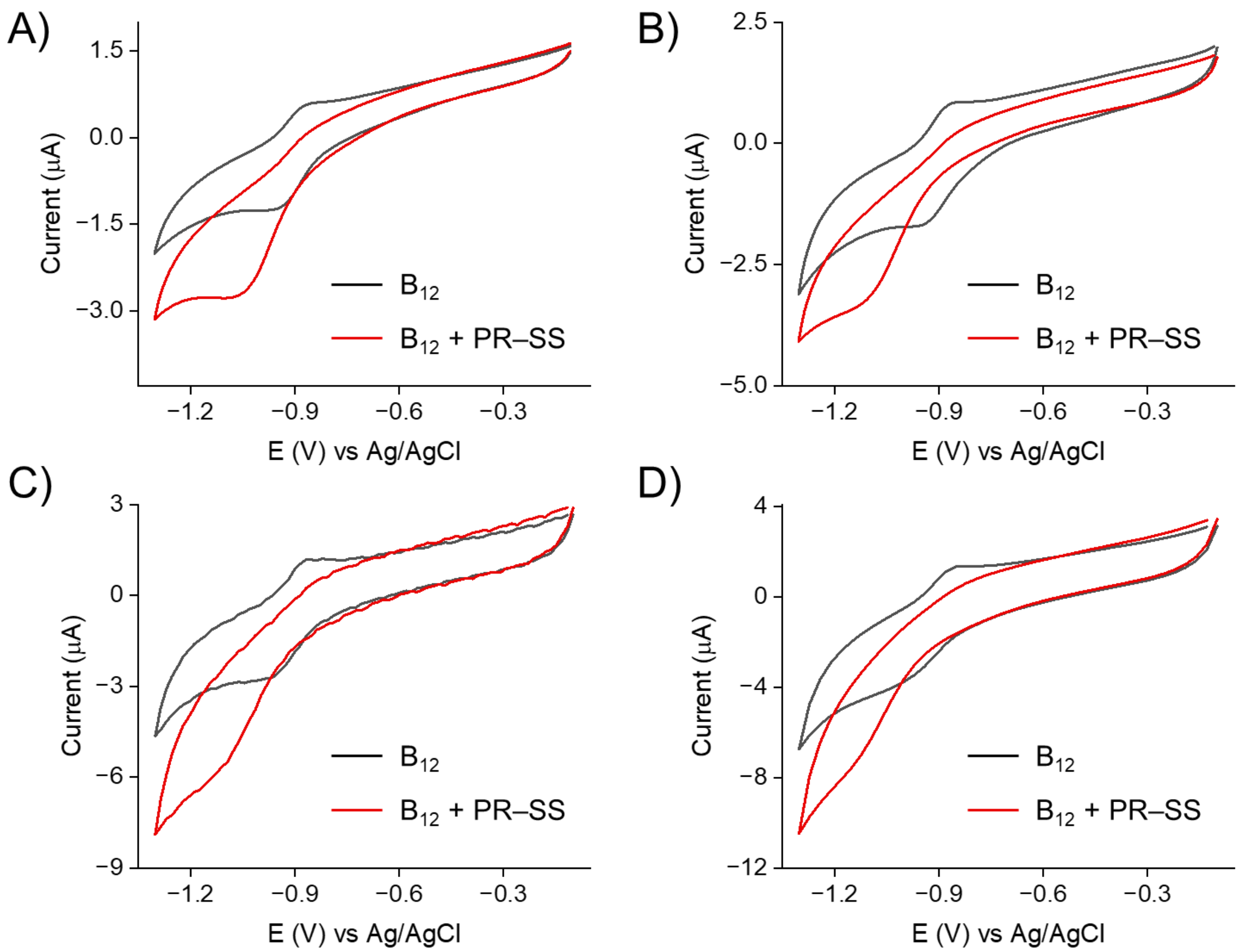

Figure S12. Cyclic voltammograms of 100 µM B_{12} solution in 0.1 M PBS (pH 7.4), under inert atmosphere in the absence (black) and presence of 50 µM PR–SS (red), recorded at glassy carbon disk electrode (Ø: 3 mm) at different scan rates (mV s^{-1}): A) 50, B) 100, C) 250 and D) 500.

4.3) Fully Electrochemical Transient Hydrogel Growth

Figure S13A shows the parallel-plate electrochemical cell configuration used in this study, consisting of a platinum counter electrode and a screen-printed electrode comprising a glassy carbon working electrode and an Ag/AgCl reference electrode. Electrochemical cells with interelectrode distances of 1, 2, 3, and 4 mm were employed. Figure S13B shows the electrochemical cell immersed in the reaction solution during the measurements.

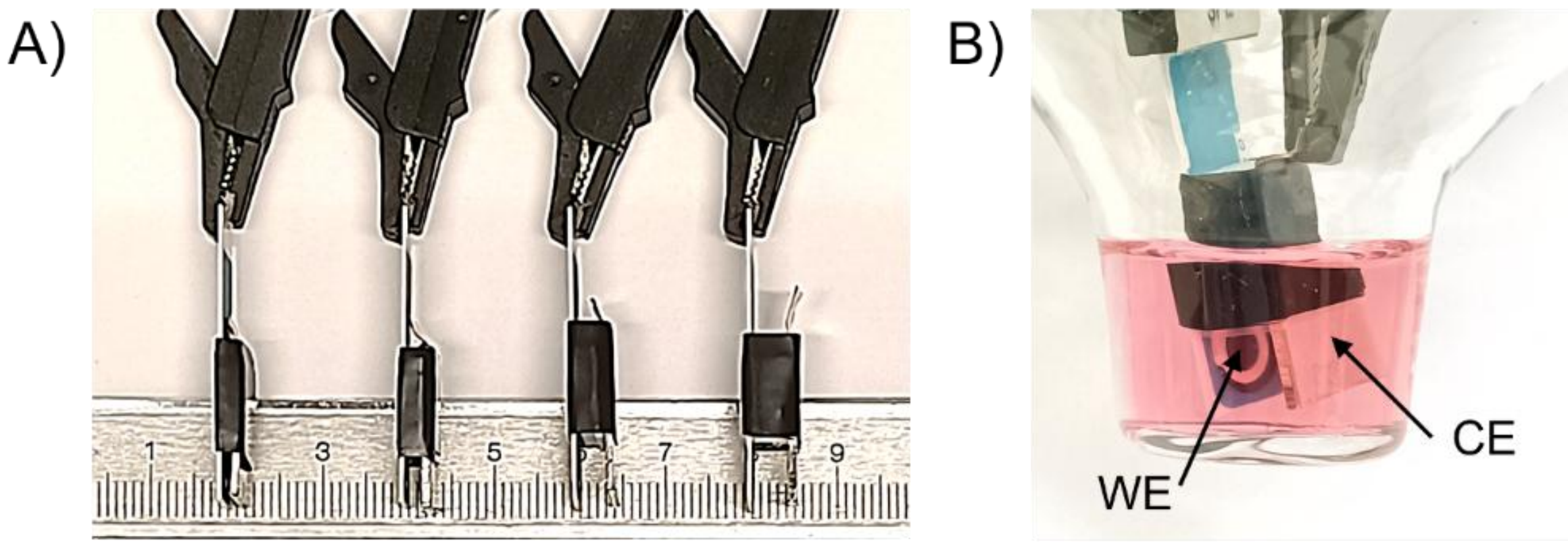

Figure S13. A) Photograph of the parallel-plate electrochemical cells with interelectrode distances of 1, 2, 3, and 4 mm (left to right). B) Photograph of the parallel-plate electrochemical cell immersed in 0.1 M PBS containing 50 µM B_{12} and 2.0 mM Fc.

Figure S14 reports the thickness of hydrogel films electrodeposited using 2 mm interelectrode distance under different applied potentials and deposition times (2–60 min) from a solution containing 4 mM Fc, 0.5 mM B_{12}, 1 mM 4-arm PEG-SH, and 1.5 mM PR–SS in 0.1 M PBS (pH 7.4), under inert atmosphere.

For all applied potentials, the film thickness initially increases with deposition time, reaches a maximum value, and subsequently decreases upon prolonged electrodeposition times. The maximum thickness, 178 µm at −0.8 V, 208 µm at −1.0 V, and 182 µm at −1.2 V, is attained at progressively shorter times as the applied potential becomes more negative, namely 20 min, 10 min, and 5 min, respectively.

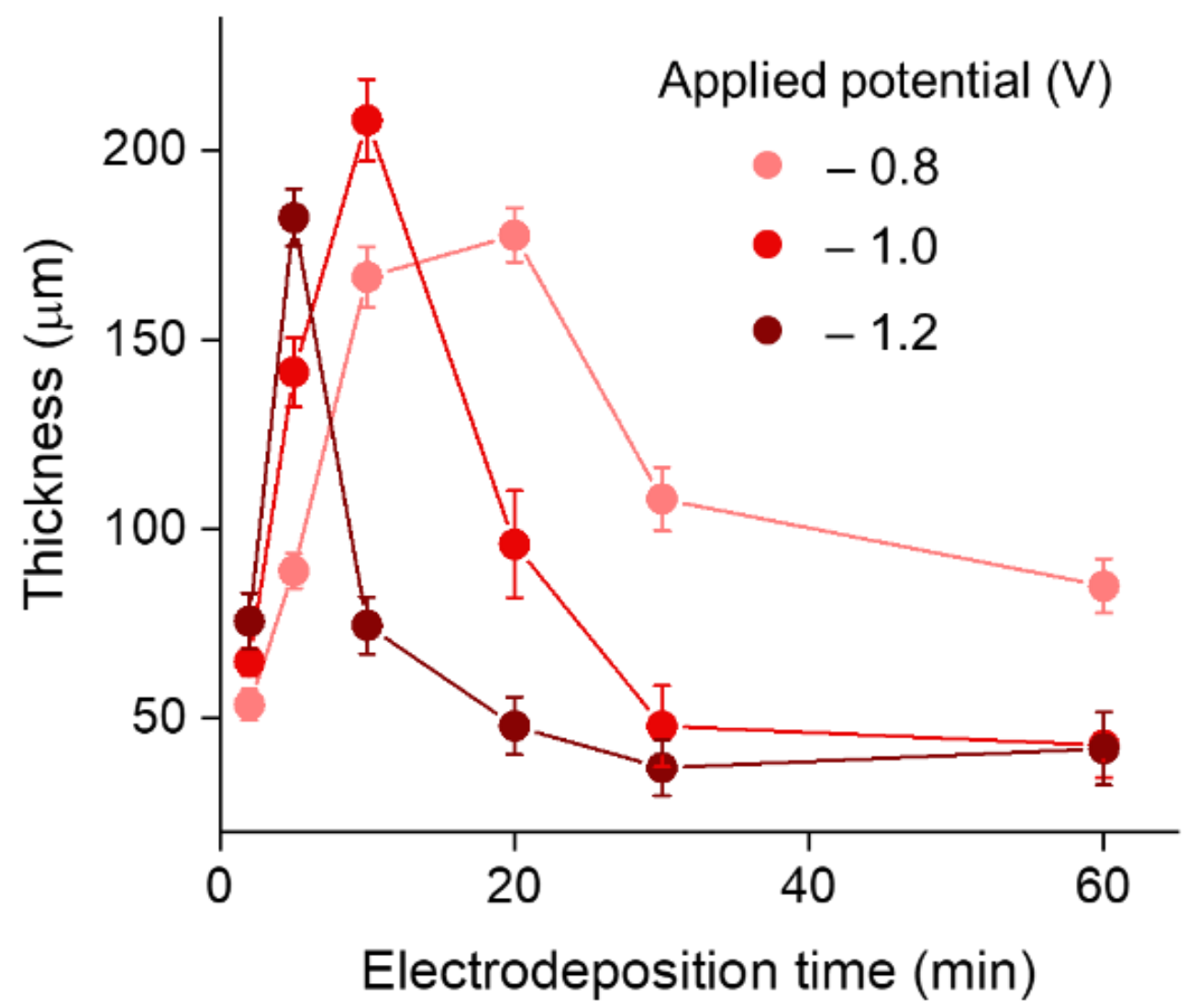

Figure S14. Thickness of hydrogel films as a function of electrodeposition time under different applied potentials (V): – 0.8, – 1.0 and – 1.2. Hydrogel films were deposited on glassy carbon electrodes (Ø: 4 mm) from a solution containing 4 mM Fc, 0.5 mM B_{12}, 1 mM 4-arm PEG-SH and 1.5 mM PR–SS, in 0.1 M PBS (pH 7.4) under inert atmosphere.

Figure S15 shows cross-sectional scanning electron microscopy (SEM) images of freeze-dried hydrogel films obtained under different electrochemical conditions using 2 mm interelectrode distance. Specifically, Figure S15A corresponds to the hydrogel formed at −0.8 V for 20 min, Figure S15B to that formed at −1.0 V for 10 min, and Figure S15C to that formed at −1.2 V for 5 min. All hydrogels exhibit a homogeneous porous morphology with a similar pore size throughout the film thickness, indicating the absence of a detectable structural gradient across the cross-section. The film thickness measured by SEM is substantially greater than that of the hydrated hydrogels owing to structural changes associated with the freeze-drying process.

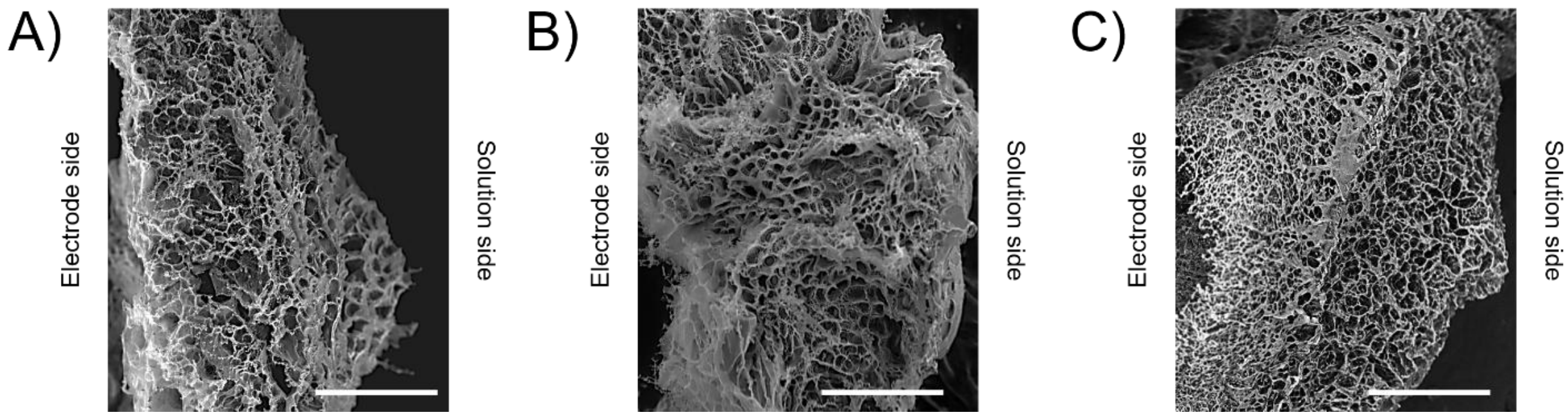

Figure S15. Cross-sectional SEM images of freeze-dried hydrogel films obtained by electrodeposition at A) −0.8 V for 20 min, B) −1.0 V for 10 min, and C) −1.2 V for 5 min. Scale bars: 0.5 mm. In each panel, the hydrogel surface in contact with the working electrode (cathode) is oriented to the left, while the surface exposed to the solution is oriented to the right.

Figure S16 shows the thickness and the amount of disulfide crosslinks for hydrogel films electrodeposited at −1.0 V for 10 min using different interelectrode distances. Electrodeposition was performed from a solution containing 4 mM Fc, 0.5 mM B_{12}, 1 mM 4-arm PEG–SH, and 1.5 mM PR–SS in 0.1 M PBS (pH 7.4), under inert atmosphere.

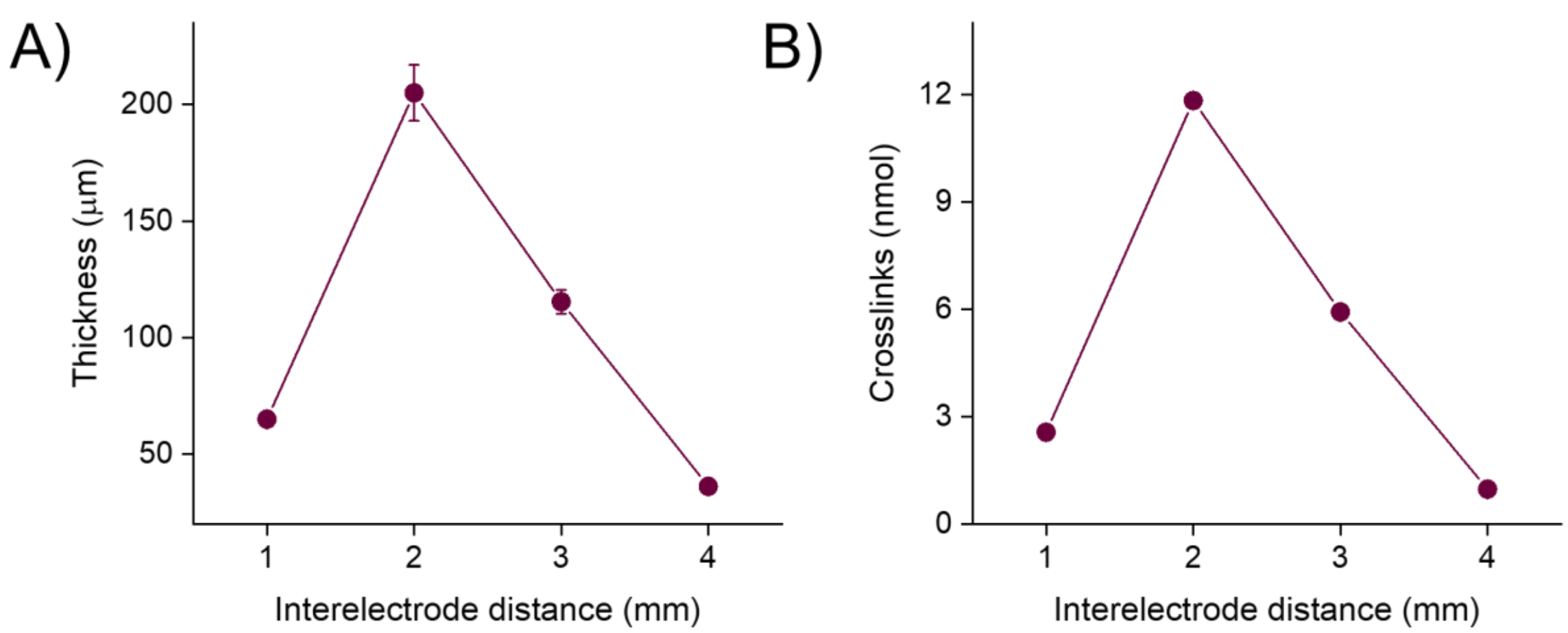

Figure S16. A) Thickness and B) amount of deposited disulfide crosslinks of hydrogel films obtained at −1.0 V for 10 min as a function of the interelectrode distance. Hydrogel films were electrodeposited from a solution containing 4 mM Fc, 0.5 mM B_{12}, 1 mM 4-arm PEG–SH, and 1.5 mM PR–SS in 0.1 M PBS (pH 7.4) under inert atmosphere.

The film thickness initially increases as the interelectrode distance is increased from 1 to 2 mm, reaching a maximum value of 205 µm, and then decreases at larger interelectrode distances (Figure S16A). No hydrogel film was deposited when a 5 mm interelectrode distance was employed. A similar trend is observed for the amount of deposited disulfide crosslinks (Figure S16B), which reaches a maximum value of 12 nmol at an interelectrode distance of 2 mm and decreases progressively as the distance is further increased.

The generation of Fc^{+} at anode in the parallel-plate electrochemical cell using 2 mm interelectrode distance, upon application of different negative potentials to the cathode, was monitored by UV–vis spectroscopy (Figure S17).

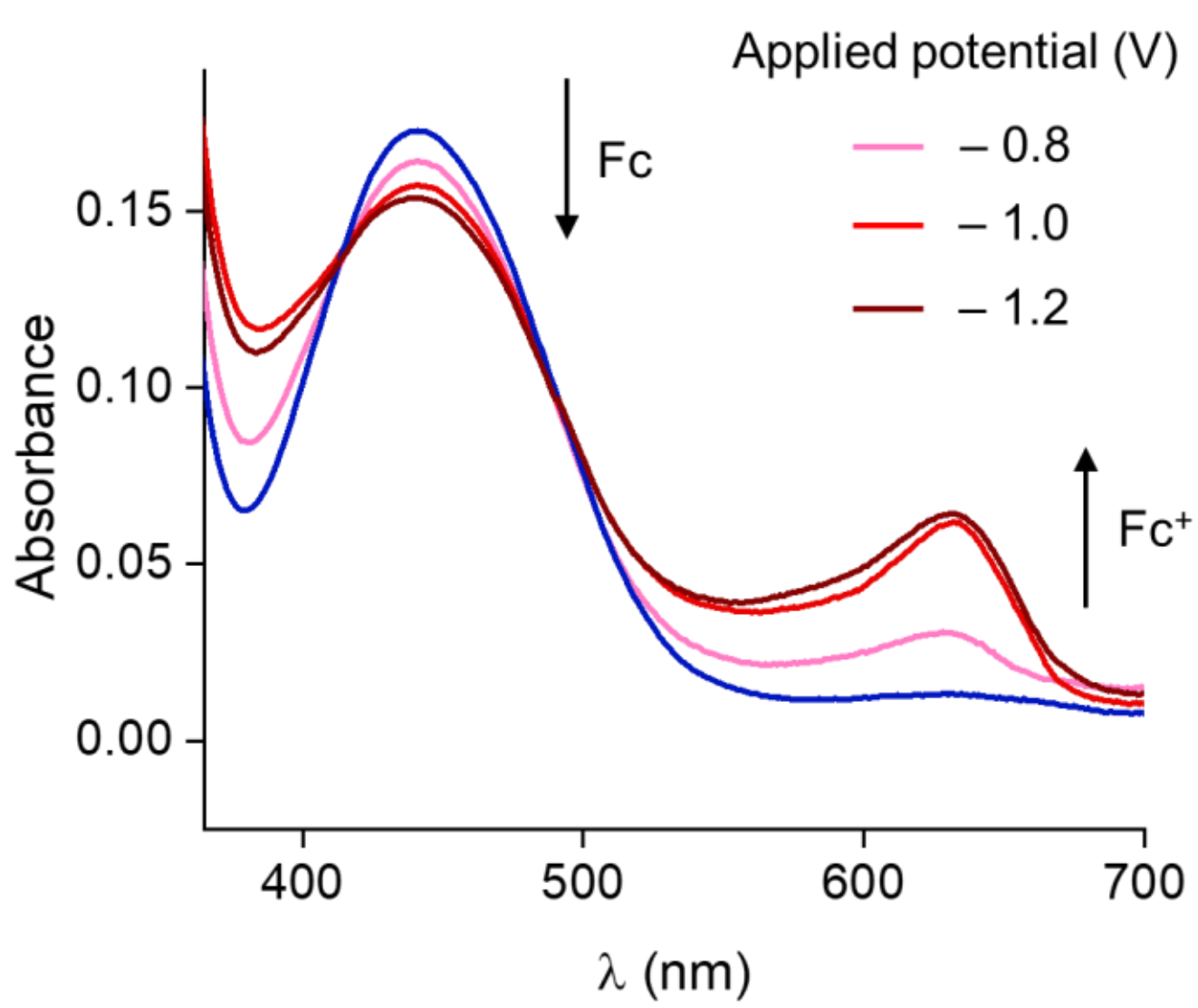

Figure S17. UV-vis spectra of a 1.5 mM Fc solution in PBS 0.1 M (pH 7.4) before (blue) and after 15 min of applied cathodic potentials (V): – 0.8 (pink), – 1.0 (red), and – 1.2 (brown).

The UV–vis spectrum of a Fc solution in the absence of an applied potential exhibits a single absorption peak at 441 nm. Upon application of −0.8 V at the cathode of the parallel-plate electrochemical cell, a decrease in the absorbance at 441 nm is observed, accompanied by the

appearance of a new peak at 631 nm, characteristic of Fc^+.[9] Applying more negative potentials at cathode results in a further decrease of the 441 nm band and a corresponding increase in the absorbance at 631 nm for the solution, indicating enhanced anodic oxidation of Fc.

4.4) Determination of Hydrogel Films Composition

The composition of the hydrogel films was determined by ^{1}H NMR spectroscopy following dissolution of the hydrogel with TCEP and expressed as the ratio between polyrotaxane and 4-arm PEG ([PR]/[4-arm PEG]). This analysis was performed by comparison with the spectra of samples of PR-SS and 4-arm PEG.

The ^{1}H NMR spectrum of PR–SS in D_2O (Figure S18A) shows two distinct sets of signals: the α-cyclodextrin (α-CD) signals at δ 4.02–3.93 and a broader signal at δ 3.92–3.48 arising from overlapping contributions of both α-CD and PEG axle protons. The α-CD signal, corresponding to 56 protons per PR molecule, was used as an internal reference due to the absence of overlapping signals. The spectrum of 4-arm PEG (Figure S18B) shows a single resonance in the region δ 3.92–3.48, characteristic of PEG protons, with no signals observed in the region δ 4.02–3.93 corresponding to α-CD. These resonances were selected for determining the hydrogel film composition because they do not overlap with the TCEP resonances, which appear at δ 2.70–2.20, consistent with the literature.[10]

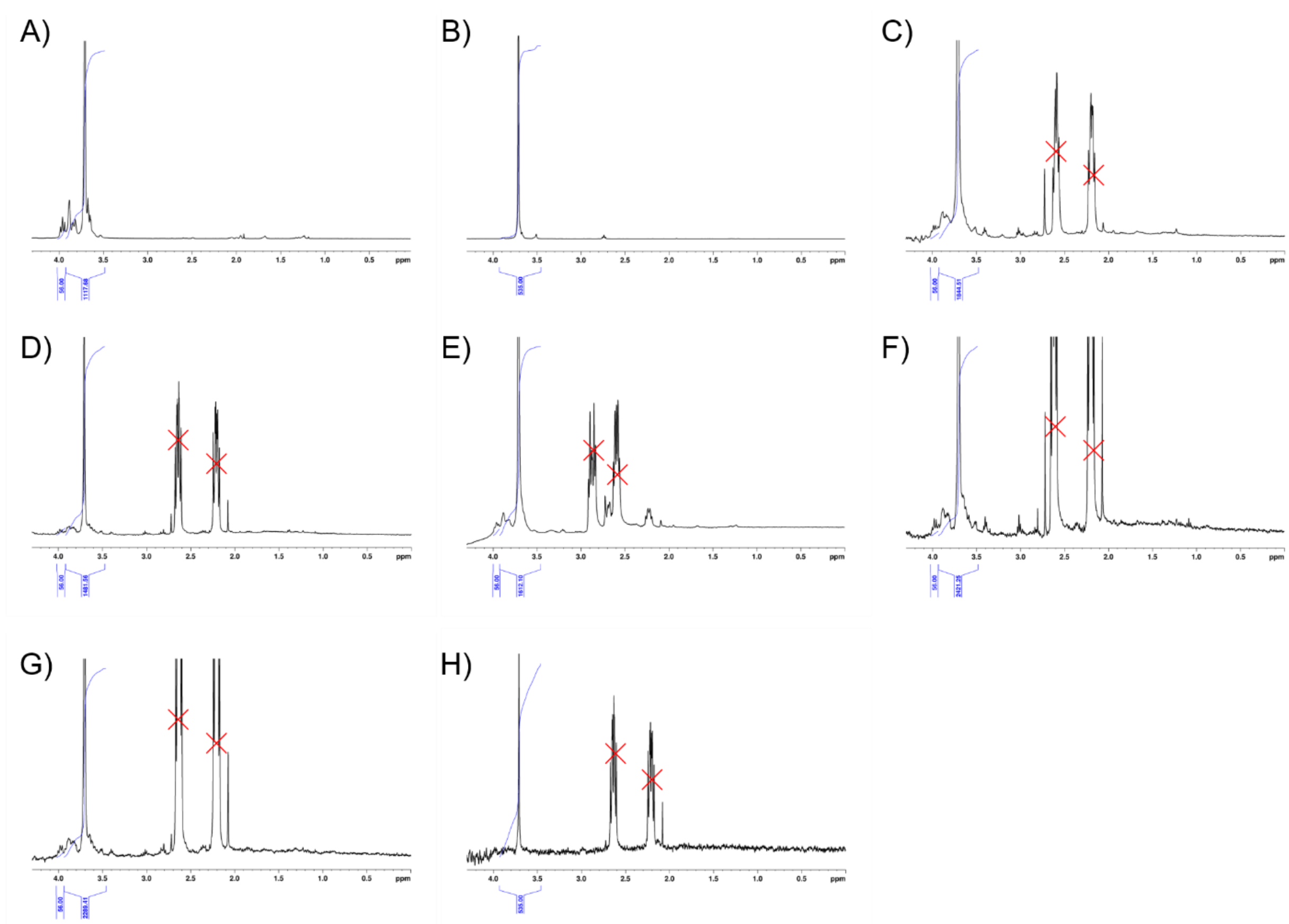

Figure S18. ^{1}H NMR spectra (400 MHz, D_2O, 298 K) of A) PR–SS and B) 4-arm PEG, and of dissolved hydrogel films electrodeposited on glassy carbon electrodes from a degassed solution containing 4 mM Fc, 0.5 mM B_{12}, 1 mM 4-arm PEG, and 1.5 mM PR–SS in 0.1 M PBS (pH 7.4), under the following conditions: C) −0.8 V for 20 min, D) −1.0 V for 5 min, E) −1.0 V for 10 min, F) −1.0 V for 20 min, and G) −1.2 V for 5 min. H) ^{1}H NMR spectrum (400 MHz, D_2O, 298 K) of a dissolved hydrogel film electrodeposited under oxidative conditions (+0.7 V for 10 min) from a degassed solution containing 4 mM Fc, 1 mM 4-arm PEG, and 1.5 mM PR–SS in 0.1 M PBS (pH 7.4), in the absence of B_{12}.

The composition of the dissolved hydrogel films was determined from the ^{1}H NMR spectrum obtained in D_2O. The [PR]/[4-arm PEG] ratio was calculated according to:

$$\frac{[PR]}{[4\ arm\ PEG]} = \frac{535}{(X - 1118)}$$

where X is the integral of the region δ 3.92–3.48 after normalization of the α-CD signal at δ 4.02–3.93 to 56 protons, corresponding to one PR unit. Under these conditions, one PR unit contributes 1118 protons to the δ 3.92–3.48 region, while the remaining integral (X−1118) arises exclusively from 4-arm PEG. The obtained value needs to be compared to 535, that corresponds to the number of protons per 4-arm PEG molecule contributing to the same region, leading to the evaluation of 4-arm PEG units relative to a single PR unit.

For the hydrogel films obtained by application of – 0.8 V for 20 min, the integral value *X* is 1845, corresponding to a [PR] / [4-arm PEG] ratio of 0.73 (Figure S18C). For films prepared at −1.0 V with varying deposition times (Figures S18D–F), the value of X increased from 1482 (5 min) to 1612 (10 min) and 2421 (20 min), corresponding to [PR]/[4-arm PEG] ratios of 1.45, 1.07, and 0.41, respectively (Figure S19).

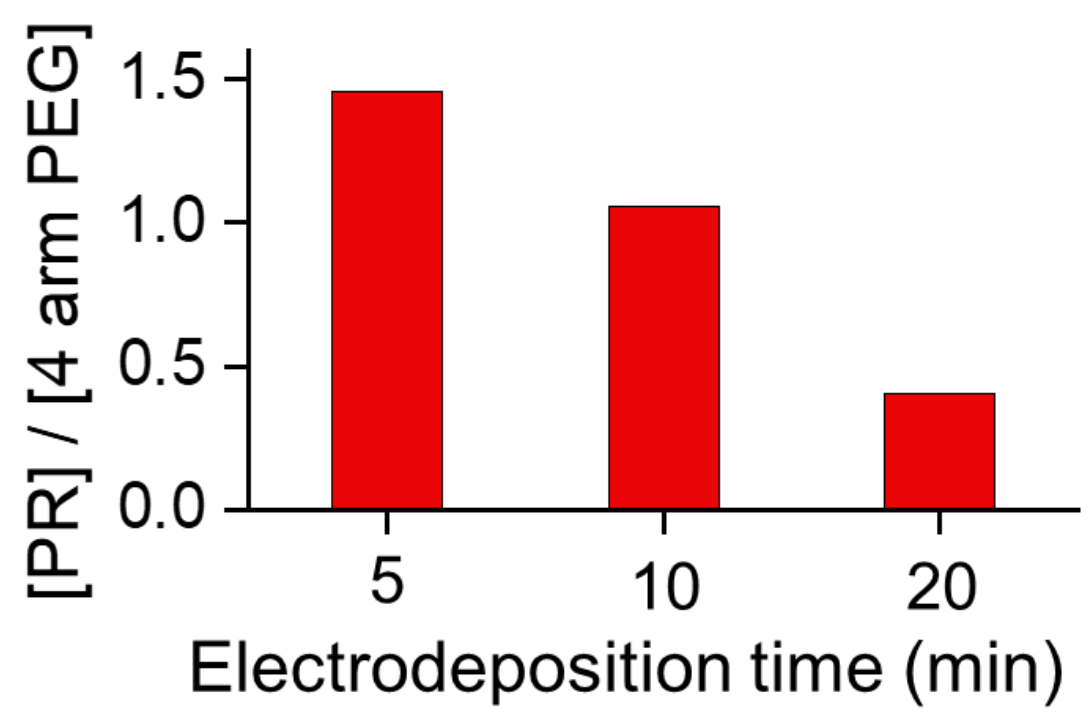

Figure S19. [PR]/[4-arm PEG] ratio in hydrogel films obtained at −1.0 V on glassy carbon electrodes from a degassed solution containing 4 mM Fc, 0.5 mM B_{12}, 1 mM 4-arm PEG, and 1.5 mM PR–SS in 0.1 M PBS (pH 7.4), as a function of deposition time.

These results indicate that longer electrodeposition times favor the formation of hydrogel films with a higher 4-arm PEG content, reflecting the combined effect of B_{12}^{-}-mediated disulfide bond cleavage and Fc^{+}-mediated disulfide reformation. For hydrogel films deposited at −1.2 V for 5 min, the integral value X was 2289, corresponding to a [PR]/[4-arm PEG] ratio of 0.45 (Figure S18G).

These results demonstrate that, under the fully electrochemical dissipative conditions, the continuous cathodic generation of B_{12}^{-} (promoting disulfide cleavage) and anodic generation of Fc^{+} (driving disulfide reformation), modulated by applied potential and time, leads to a progressive variation in the chemical composition of the hydrogel films. This reflects dynamic network depolymerization and re-crosslinking processes.

4.5) Electrodeposition of Hydrogel Films Under Equilibrium Conditions

Figure S20 displays the thickness, total number of disulfide cross-links, and cross-link densities (ν) of hydrogel films deposited under an oxidative potential (+0.7 V) from a solution containing 4 mM Fc, 1 mM 4-arm PEG, and 1.5 mM PR-SS in 0.1 M PBS (pH 7.4), in the absence of B_{12}.

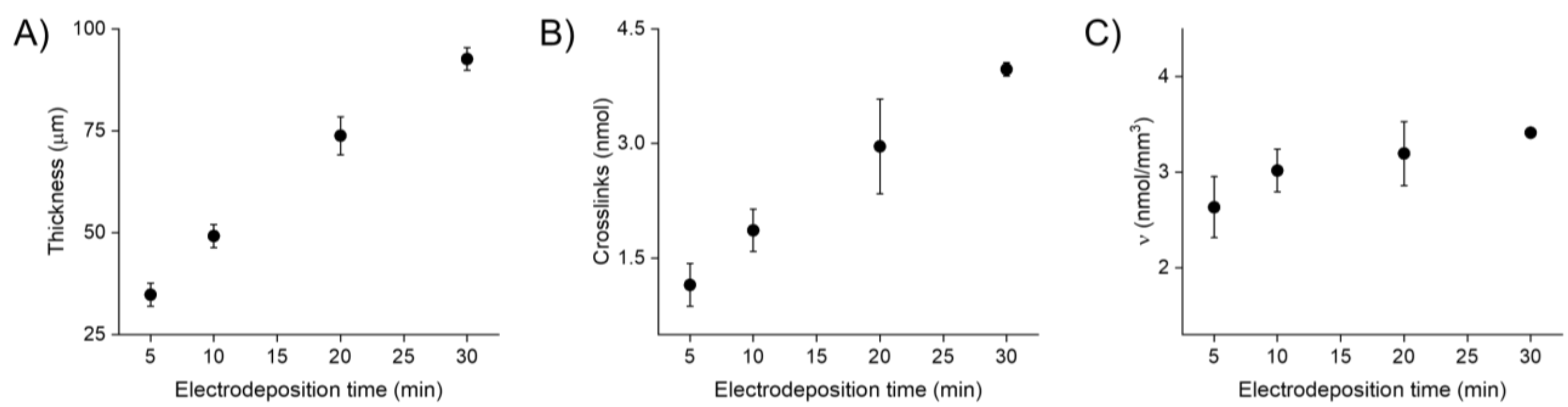

Figure S20. A) Film thickness, B) total number of disulfide cross-links, and C) cross-link density (ν) of hydrogel films obtained on glassy carbon electrodes from a degassed solution containing 4 mM Fc, 1 mM 4-arm PEG, and 1.5 mM PR–SS in 0.1 M PBS (pH 7.4), under an applied potential of +0.7 V, as a function of deposition time.

The thickness (Figure S20A) and the total number of cross-links (Figure S20B) of the obtained films increase linearly with deposition time. The cross-link density (Figure S20C) remains essentially constant (~3.0 nmol mm^{-3}), independent of the electrodeposition duration. These results

highlight the essential role of B_{12} in establishing an antagonistic redox mediation process that underlies the transient growth of the hydrogel network.

4.6) Electrodeposition of Control Hydrogel Films (8-Arm PEG-SS)

Control hydrogel films were electrodeposited using 8-arm PEG-SS in place of PR–SS under applied potentials of −0.8 V for 20 min, −1.0 V for 10 min, and −1.2 V for 5 min. The deposition was performed from a degassed solution containing 4 mM Fc, 0.5 mM B_{12}, 1 mM 4-arm PEG, and 1.3 mM 8-arm PEG-SS, using 2 mm interelectrode distance (Figure S21).

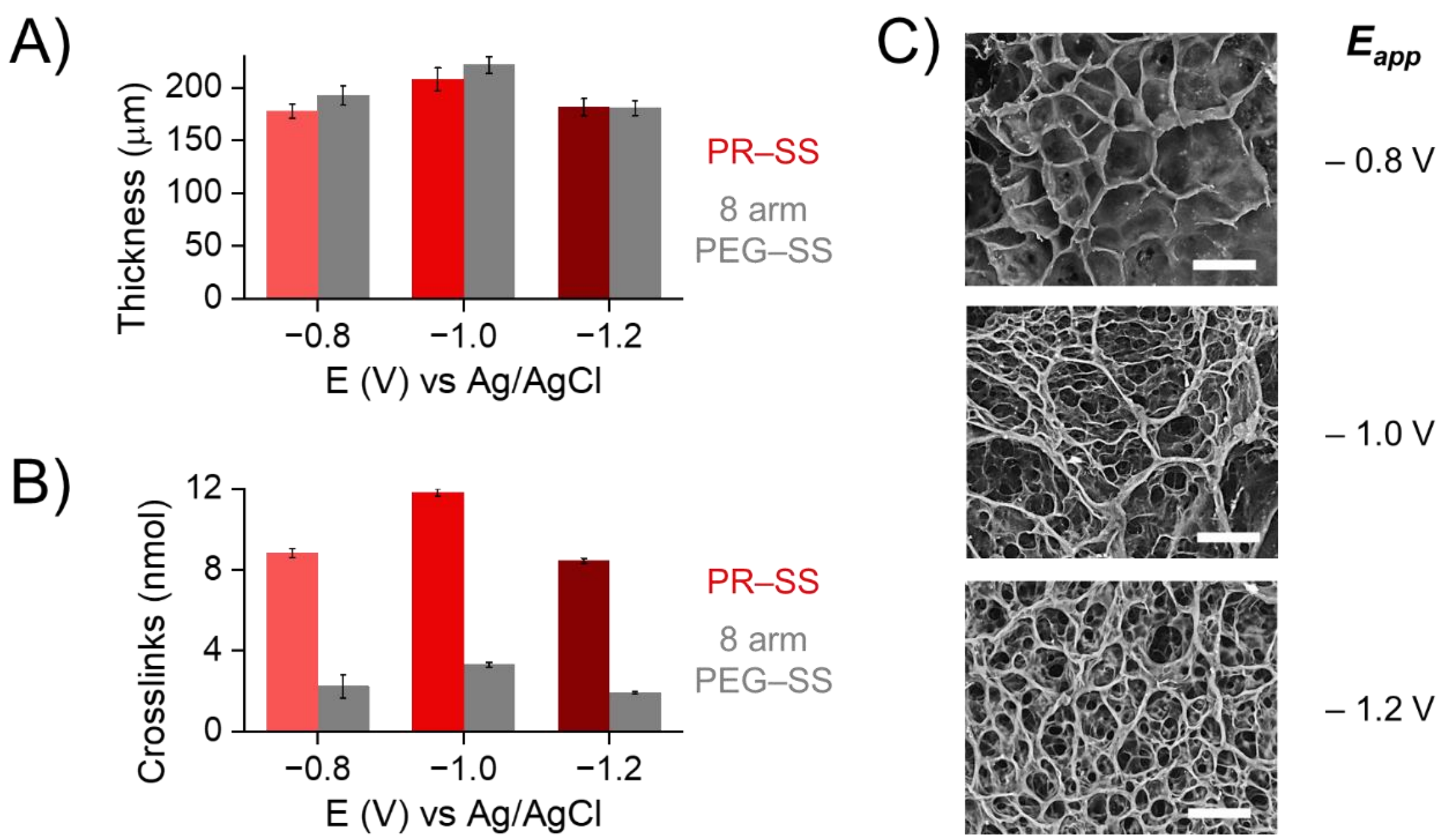

Figure S21. A) Thickness and B) total number of disulfide crosslinks as a function of the applied potential for hydrogel films electrodeposited on glassy carbon electrodes (Ø = 4 mm) from a degassed solution containing 0.5 mM B_{12}, 4 mM Fc, and 1 mM 4-arm PEG-SH in 0.1 M PBS (pH 7.4), in the presence of 1.5 mM PR–SS (red) or 1.3 mM 8-arm-PEG-SS (gray). C) SEM images of 8-arm PEG-SS-based hydrogel films obtained at different applied potentials (scale bar: 50 μm).

The thickness of the obtained hydrogel films (Figure S21A) reaches a maximum value of 222 μm for deposition at −1.0 V, followed by 193 μm at −0.8 V and 181 μm at −1.2 V. These thickness

values are comparable to those obtained for films electrodeposited from PR–SS under identical conditions. The total number of disulfide cross-links (Figure S21B), reaches a maximum value of 3.31 nmol at −1.0 V, followed by 2.24 nmol at −0.8 V and 1.93 nmol at −1.2 V. These values are approximately fourfold lower than those measured for PR–SS-based films under the same conditions. SEM images of the freeze-dried 8-arm PEG-SS-based hydrogel films obtained at different deposition potentials reveal a three-dimensional porous network structure characteristic of hydrogels (Figure S21C).

4.7) Rheological Characterization of Hydrogel Films

Rheological characterization of hydrogel films prepared via the fully electrochemical dissipative fabrication strategy is shown in Figure S22. Films were electrodeposited from a degassed solution containing 0.5 mM B_{12}, 4 mM Fc, and 1 mM 4-arm PEG in 0.1 M PBS (pH 7.4), in the presence of either 1.5 mM PR–SS or 1.3 mM 8-arm PEG-SS (control), under different applied potentials. Figure S22A shows the frequency sweep (1% strain) for hydrogel films deposited at −1.0 V for 10 min. The polyrotaxane (PR–SS)-based film exhibits an elastic modulus (*G'* = 21 ± 2 Pa) significantly higher than that of the 8-arm PEG-SS-based film (G′ = 2 ± 1 Pa), consistent with a higher cross-linking density of the network. Moreover, the PR–SS-based hydrogel maintains viscoelastic solid-like behavior (G′ > G″) over a broader frequency range compared to the 8-arm PEG-SS film, for which the crossover of G′ and G″ occurs at 40 Hz and 13 Hz, respectively. This behavior suggests additional energy dissipation arising from the sliding ring dynamics within the polyrotaxane network.[2]

Similar trends are observed for the rheological characterizations of hydrogel films deposited at –0.8 V for 20 min, across the tested frequency range at 1 % strain (Figure S22B) and over the strain range at 1 Hz (Figure S22D). In particular, PR–SS-based networks exhibit significantly higher *G'*

values (15 ± 2 Pa) compared to 8-arm PEG-SS-based films (2 ± 1 Pa). Moreover, the PR–SS hydrogels maintain viscoelastic solid-like behavior (G′ > G″) over a broader range of strain and frequency, with G′-G″ crossover points at 630% strain and 30 Hz, respectively, compared to 250% strain and 13 Hz for the 8-arm PEG-SS films.

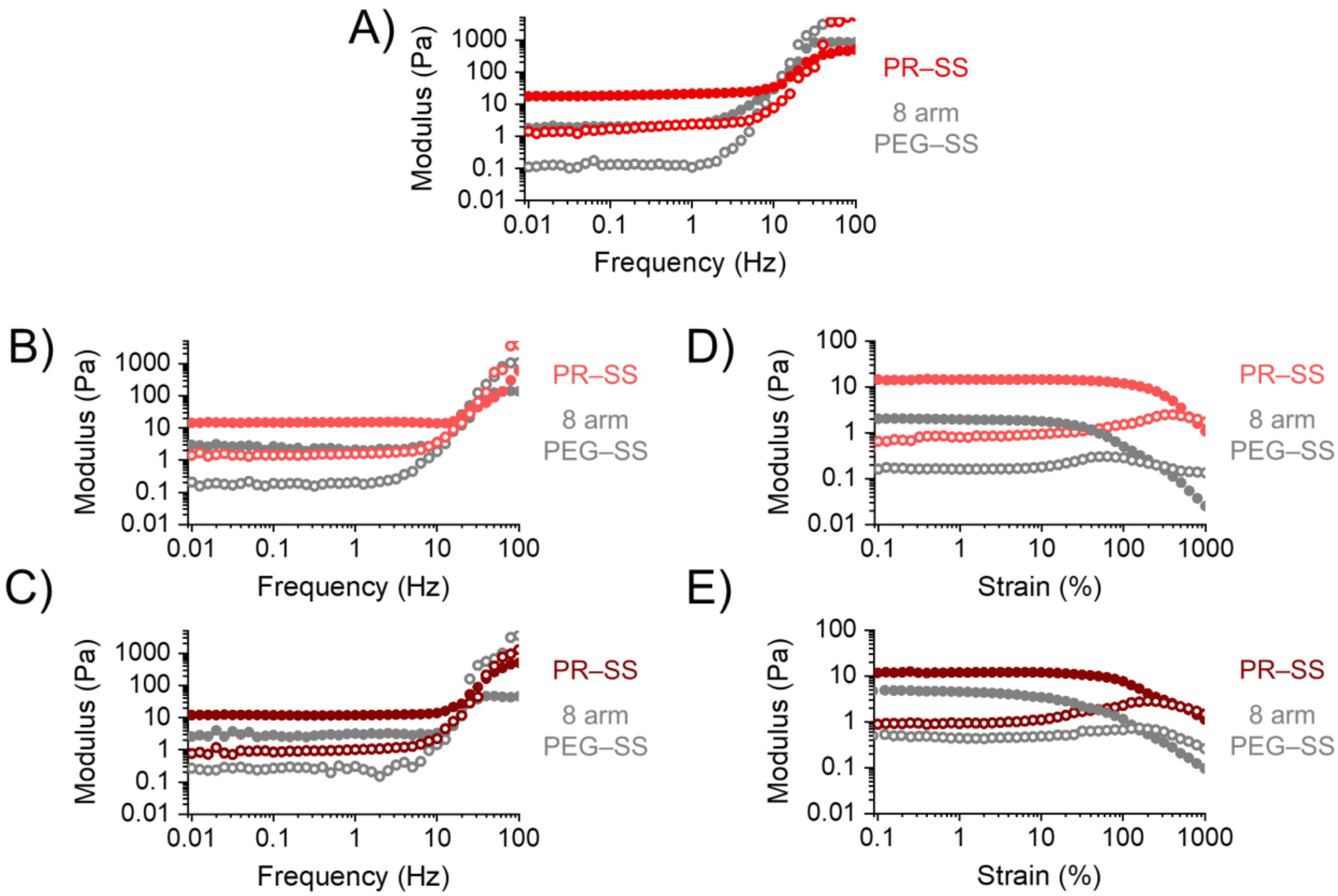

Figure S22. A–C) Frequency sweeps recorded at 1 % strain for hydrogel films electrodeposited from a degassed solution containing 0.5 mM B_{12}, 4 mM Fc, and 1 mM 4-arm PEG in 0.1 M PBS (pH 7.4), in the presence of 1.5 mM PR–SS (red) or 1.3 mM 8-arm PEG-SS (gray), at A) −1.0 V for 10 min, B) −0.8 V for 20 min, and C) −1.2 V for 5 min. D–E) Strain sweeps recorded at 1 Hz for hydrogels films prepared under the same conditions in the presence of 1.5 mM PR–SS (red) or 1.3 mM 8-arm PEG-SS (gray) at D) −0.8 V for 20 min and E) −1.2 V for 5 min. Filled symbols represent G′, and open symbols represent G″.

A similar trend is observed for hydrogel films deposited at −1.2 V for 5 min (Figures S22C and S22E). The PR-SS-based networks exhibit higher G′ values (12 ± 1 Pa) than the 8-arm PEG-SS based films (4 ± 2 Pa) and retain viscoelastic behavior over a wider range of strain and frequency,

with G′-G″ crossover points at 500% strain and 40 Hz, respectively, compared to 150% strain and 15 Hz for the control films.

5. Fully Electrochemical Transient Hydrogel Softening

Figure S23 shows the temporal evolution of the storage modulus (*G'*) of an 8-arm PEG-SS based hydrogel-coated electrode (prepared according to our approach (see *Experimental Section Paragraph 4.2*)), during the application of −1.0 V in degassed 0.1 M PBS (pH 7.4) containing 50 μM B_{12} and 2 mM Fc. A progressive decrease in overall hydrogel stiffness is observed, with G′ decreasing from approximately 2 Pa before voltage application to 0.3 Pa after 60 min. Longer time of voltage application results in extensive weakening of the hydrogel network, ultimately leading to film detachment from the electrode surface.

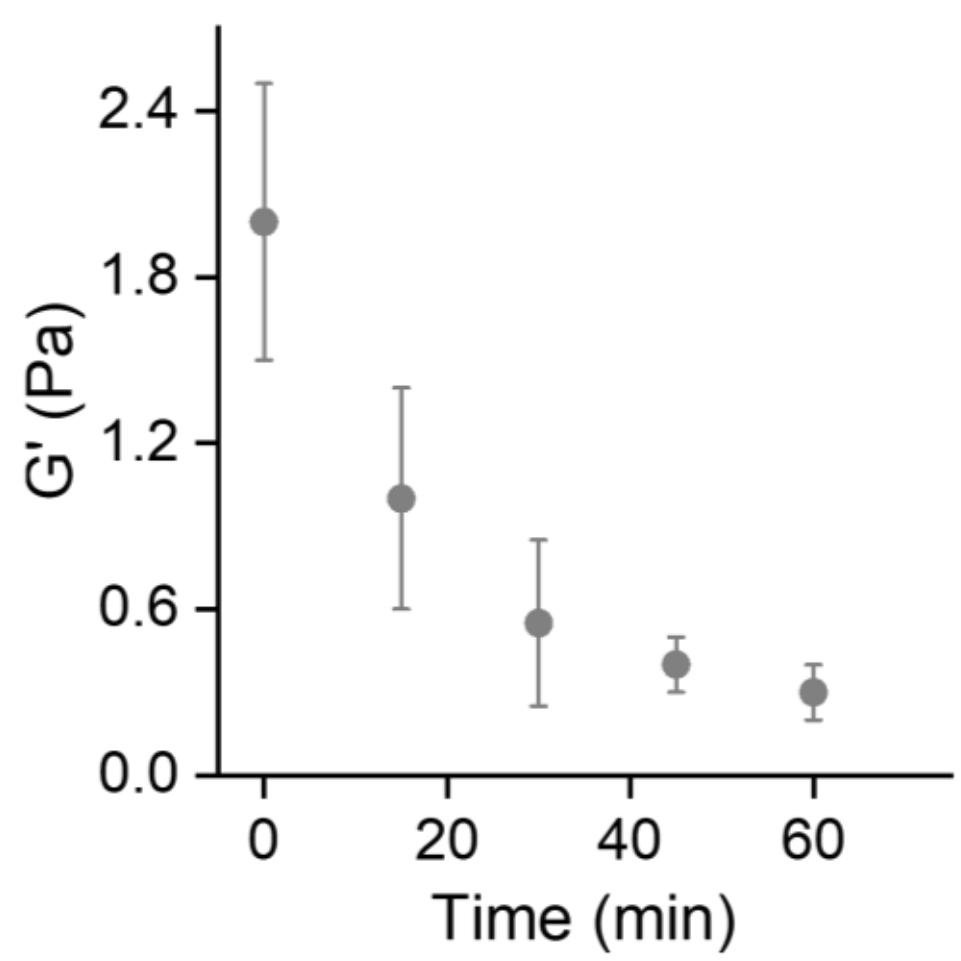

Figure S23. Temporal changes in stiffness (*G'*) of 8-arm PEG-SS-based hydrogel under application of −1.0 V in a 50 μM B_{12} degassed solution in PBS 0.1 M (pH 7.4) in the presence of 2 mM Fc.

Figure S24A shows the chronoamperometric curves recorded for a PR-SS-based hydrogel-coated electrode subjected to −1.0 V in degassed 0.1 M PBS (pH 7.4) containing 50 μM B_{12} and increasing concentrations of Fc. In all cases, the cathodic current gradually decreases with time, reaching a

near-steady-state value after approximately 120 min. As the Fc concentration increases, the steady-state current decreases slightly, from −1.3 μA at 0.5 mM Fc to −0.70 μA at 8.5 mM Fc. The charge obtained from the integration of chronoamperometric curves (Figure S24B) similarly decreases from −14.5 mC at 0.5 mM Fc to −10.0 mC at 8.5 mM Fc. This trend is attributed to the higher concentration of oxidizing mediator, which promotes oxidation reactions in the bulk hydrogel matrix, thereby reducing the net cathodic current and the total charge passed during the experiment.

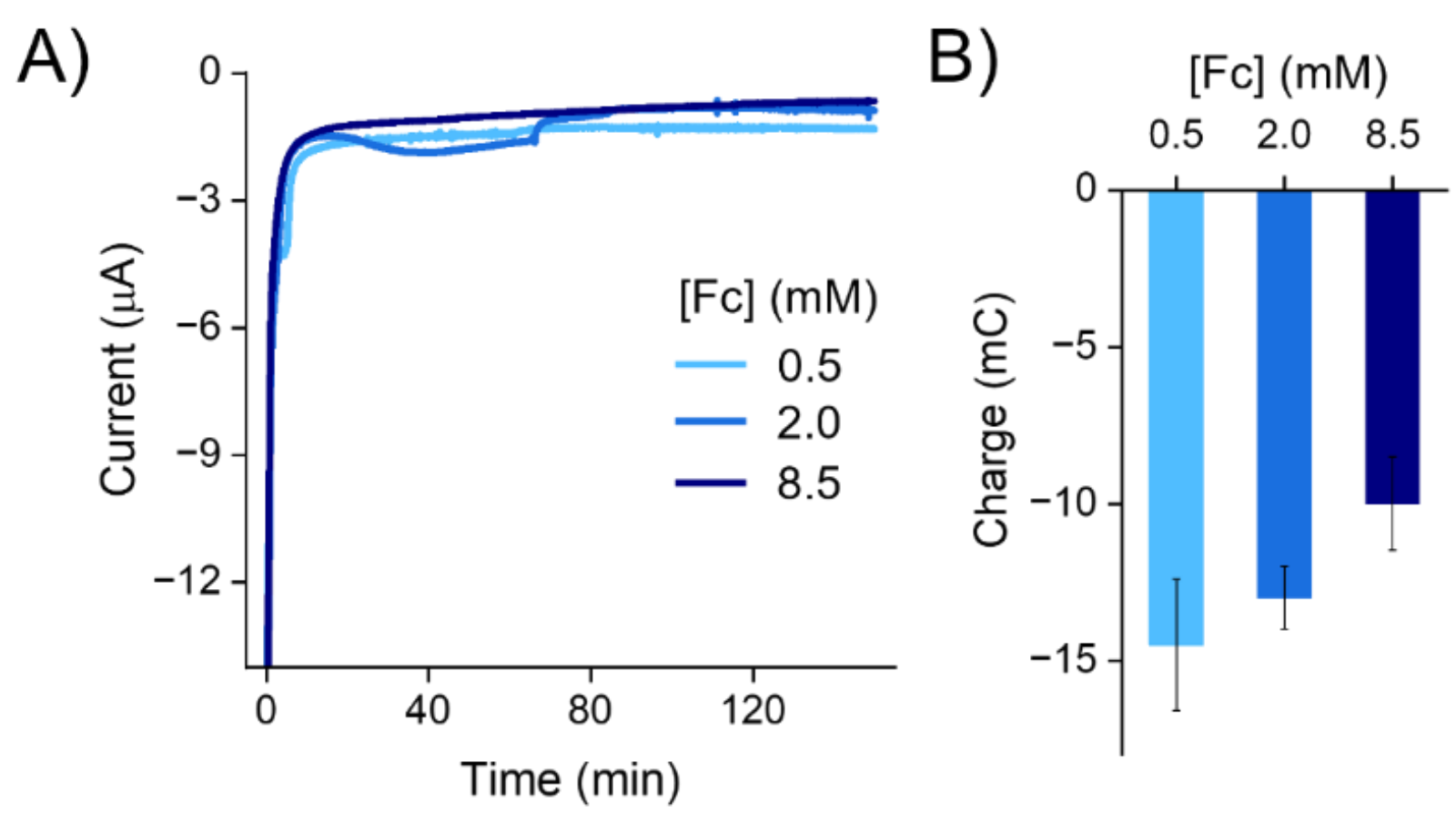

Figure S24. A) Chronoamperometric curves recorded during the transient softening of PR-SS-based hydrogel films on glassy carbon electrodes under an applied potential of −1.0 V in degassed 0.1 M PBS (pH 7.4) containing 50 μM B_{12} and increasing concentrations of Fc (0.5, 2.0, and 8.5 mM). B) Integrated charge passed during the antagonistic redox cycle at an applied potential of −1.0 V as a function of Fc concentration.

Electrochemical impedance spectroscopy (EIS) was employed to monitor the electrochemical-induced softening of the hydrogel network (Figure S25). Measurements were performed on PR-SS-based hydrogel-coated electrodes in a parallel-plate electrochemical cell at −1.0 V in degassed 0.1 M PBS (pH 7.4) containing 50 μM B_{12} and different concentrations of Fc (0.5, 2.0, 8.5 mM).

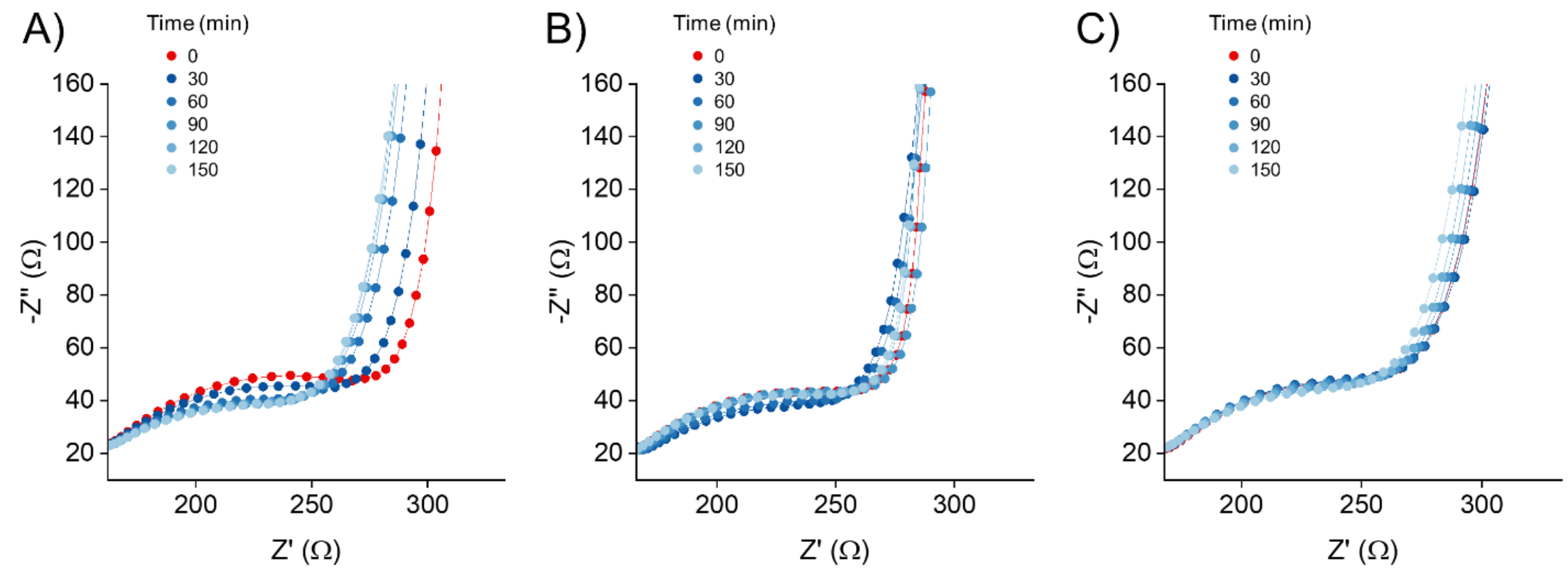

Figure S25. Nyquist plots of PR-SS-based hydrogel-modified electrodes in the presence of 50 µM B_{12} and varying concentrations of Fc: A) 0.5 mM, B) 2.0 mM, and C) 8.5 mM.

The formation of thiols during electrochemically induced softening of the PR-SS-based hydrogel was monitored by confocal fluorescence microscopy using fluorescein isothiocyanate (FITC) as a probe. Hydrogel-coated electrodes were immersed in 0.1 M PBS (pH 7.4) containing 50 µM B_{12} and varying concentrations of Fc, and a potential of −1.0 V was applied. Confocal fluorescence images were collected prior to voltage application, after 30 min (corresponding to the minimum stiffness at intermediate Fc concentration, observed by rheology), and after 150 min (Figure S26A).

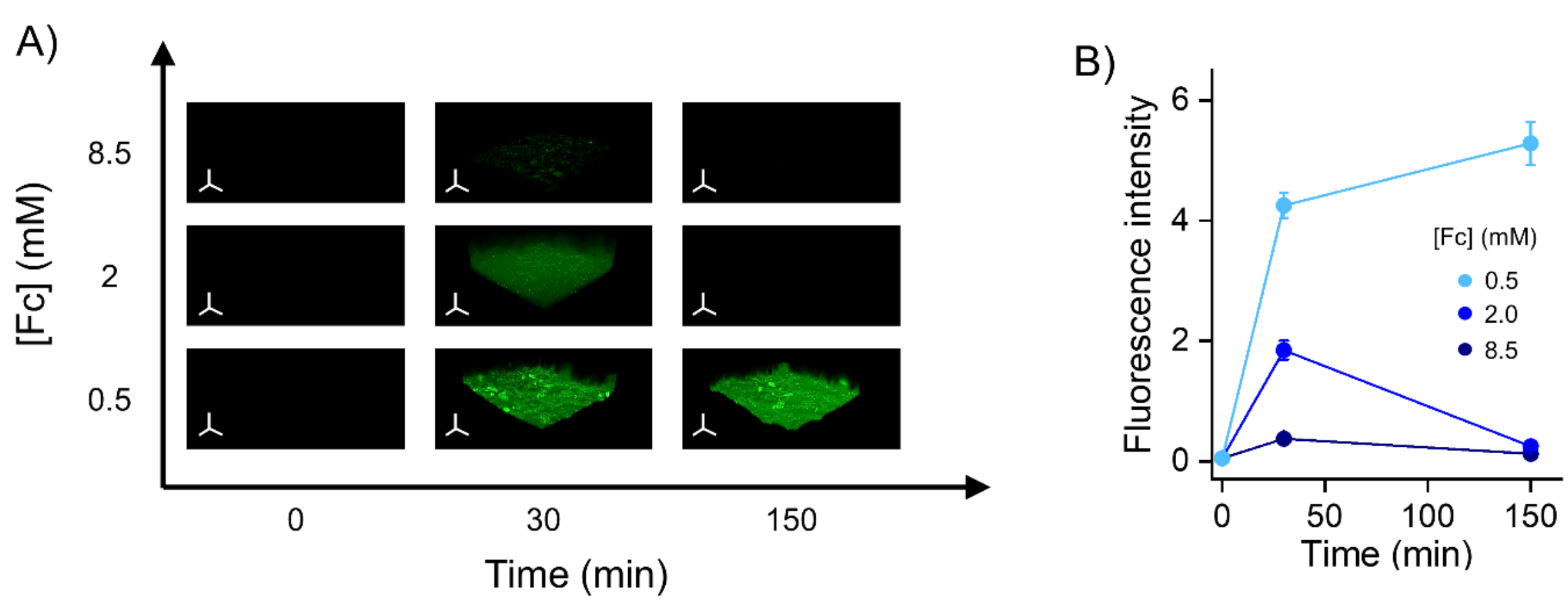

Figure S26. A) Laser scanning confocal microscopy images of fluorescently labeled PR-SS-based hydrogel films acquired after 30 and 150 min of −1.0 V potential application under inert atmosphere in 0.1 M PBS (pH 7.4) containing 50 µM B_{12} and different concentrations of Fc (scale bar: 50 µm). B) Fluorescence intensity collected under voltage application at different Fc concentrations.

At 0.5 mM Fc, the fluorescence intensity increases markedly and remains constant over time. At 2.0 mM Fc, the fluorescence intensity increases after approximately 30 min and subsequently decreases to its initial value. At 8.5 mM Fc, no significant fluorescence is detected during voltage application, indicating that the network remains in a highly cross-linked state.

6. Bioelectrochemically Controlled Insulin Release

6.1) (Bio)-Electrochemical Characterization

Figure S27A shows the CVs performed at electrodes modified with protein-loaded PR–SS hydrogel films in 0.1 M PBS (pH 7.4) containing 50 μM B_{12} and 8.5 mM Fc, upon addition of increasing concentrations of glucose under inert atmosphere. The potential window (−0.1 V to +0.6 V) was selected to probe only the Fc redox processes while avoiding electron transfer involving B_{12}. In the absence of glucose, a pair of anodic and cathodic peaks is observed at 301 mV and 221 mV, respectively, with comparable current intensities, characteristic of Fc redox behavior. Upon increasing the glucose concentrations, a progressive increase in the anodic current accompanied by a decrease in the cathodic current is observed. This behavior indicates that Fc^+ participates in glucose oxidation, acting as a redox mediator for glucose oxidase embedded within the hydrogel network. Chronoamperometric measurements performed at an applied potential of +0.8 V show a stepwise increase in current upon successive addition of glucose (Figure S27B). The current response exhibits a linear dependence on glucose concentration up to 20 mM (Figure S27C).

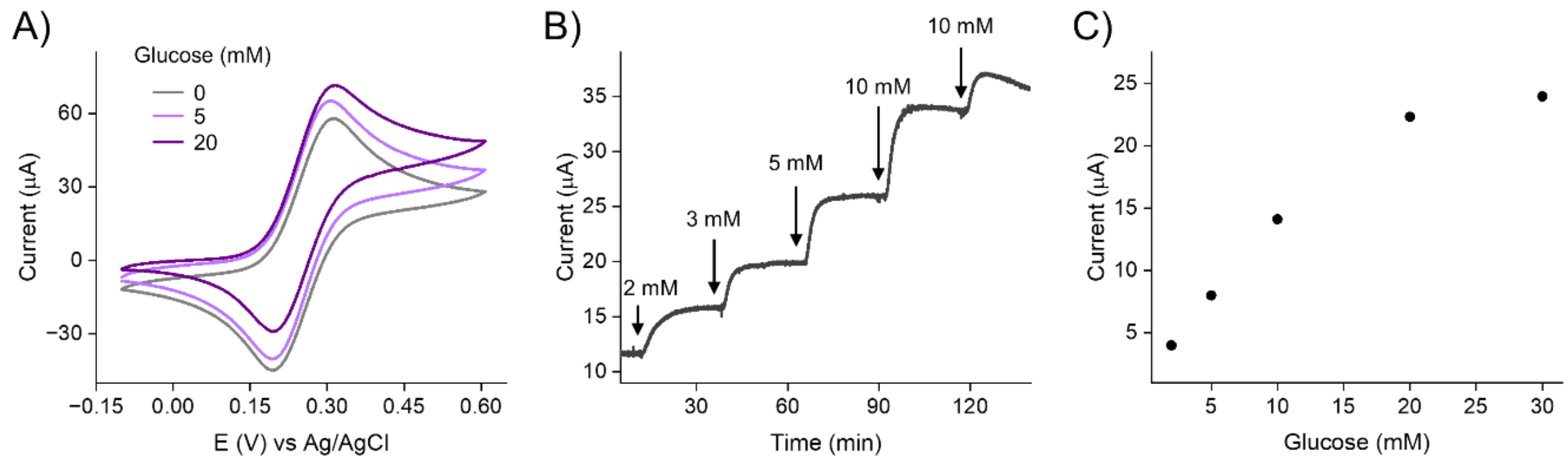

Figure S27. A) Cyclic voltammograms (10 mV s^{-1}) recorded under inert atmosphere at working electrodes modified with protein-loaded PR-SS-based hydrogel films in 0.1 M PBS (pH 7.4) containing 50 µM B_{12} and 8.5 mM Fc, in the presence of increasing concentrations of glucose: 0, 5, and 20 mM. B) Chronoamperometric response at +0.8 V (vs. Ag/AgCl) upon successive additions of glucose. C) Dependence of the current on the glucose concentration.

6.2) Release of FITC-Insulin from Protein-Loaded Hydrogels

Figure S28 shows the cumulative release profile from electrode coated with protein-loaded PR-based hydrogel films, incubated in 0.1 M PBS (pH 7.4) containing 50 µM B_{12} and subjected to an applied potential of -1.0 V under inert atmosphere.

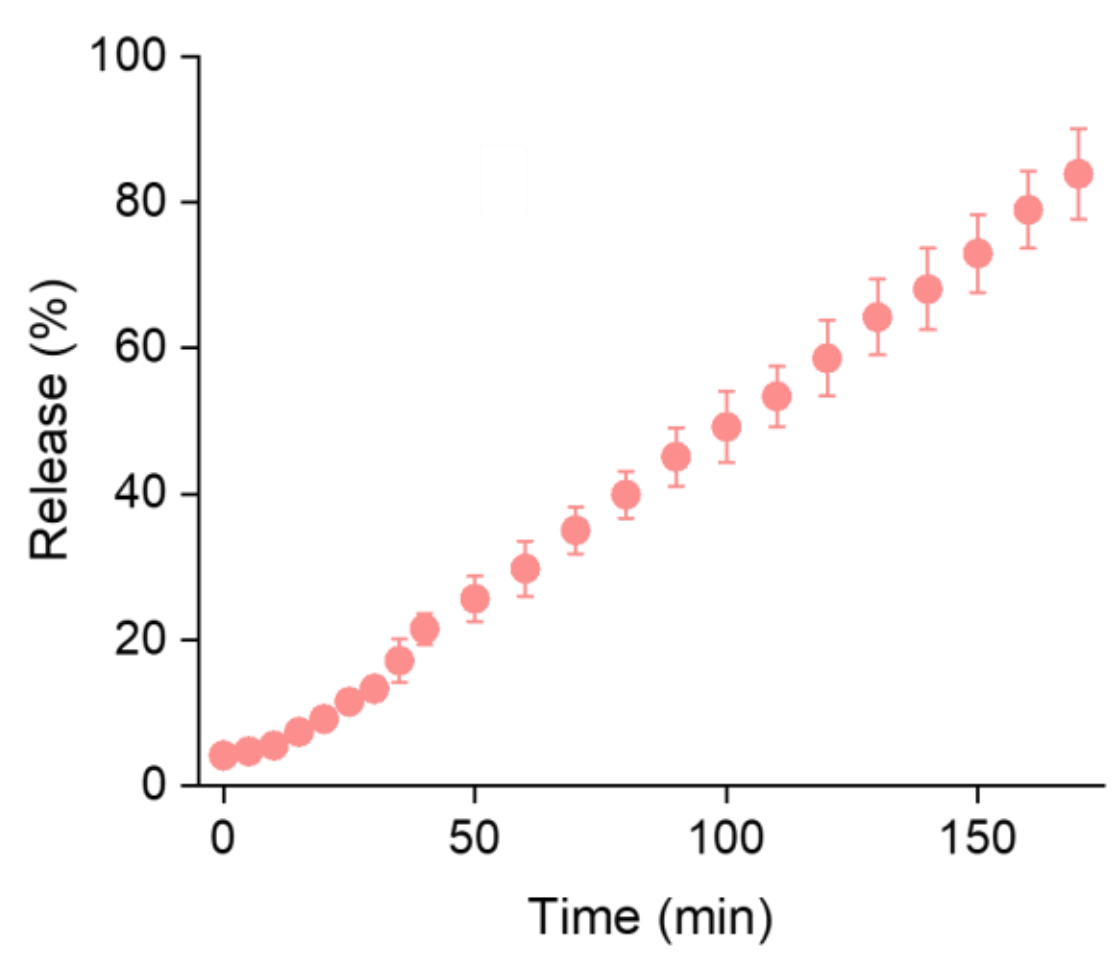

Figure S28. Release of FITC-insulin from electrodes modified with protein-loaded PR-SS-based hydrogel films, incubated in 0.1 M PBS (pH 7.4) containing 50 µM B_{12}, under an applied potential of −1.0 V in inert atmosphere.

The cumulative FITC-insulin release reaches 84 ± 6% after 170 min, significantly higher than that observed in the presence of both Fc and glucose. These results indicate that B_{12}-mediated disulfide cleavage induces progressive network weakening, resulting in rapid and near-complete release.

The time-dependent cumulative release from electrodes coated with protein-loaded hydrogel films based on control 8-arm PEG-SS was then investigated. The hydrogel-modified electrodes were incubated in 0.1 M PBS (pH 7.4) containing 50 µM B_{12} and 8.5 mM Fc and subjected to an applied potential of -1.0 V under inert atmosphere. In the absence of glucose, a progressive release of FITC-insulin is observed, reaching 37 ± 4% after 170 min (Figure S29). Upon addition of 5 mM glucose, a modest increase in release is observed, reaching 45 ± 4% at the same time point. Increasing the glucose concentration to 20 mM results in only a slight additional increase, reaching 50 ± 4% after 170 min. These results indicate that FITC-insulin release from 8-arm PEG-based hydrogel films is not significantly regulated by glucose.

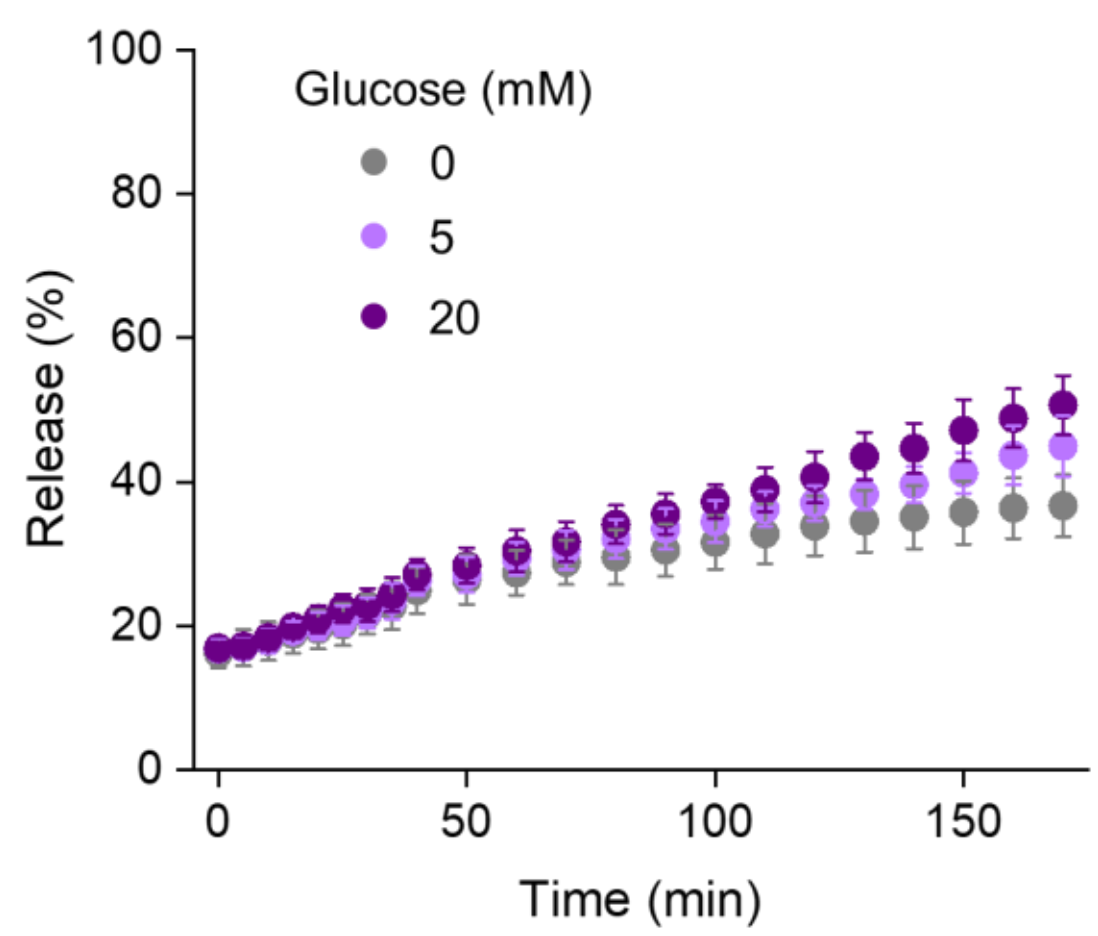

Figure S29. Release of FITC-insulin from electrodes modified with protein-loaded 8-arm PEG-based hydrogel films, incubated in 0.1 M PBS (pH 7.4) containing 50 µM B_{12} and 8.5 mM Fc, under an applied potential of −1.0 V in inert atmosphere, in the presence of increasing glucose concentrations: 0 mM (gray), 5 mM (pink), and 20 mM (purple).

7. References

[1] Zugates, G. T.; Anderson, D. G.; Little, S. R.; Lawhorn, I. E. B.; Langer, R. *J. Am. Chem. Soc.* **2006**, *128*, 12726–12734.

[2] Jiang, Y.; Zhang, Z.; Wang, Y.-X.; Li, D.; Coen, C.-T.; Hwaun, E.; Chen, G.; Wu, H.-C.; Zhong, D.; Niu, S.; Wang, W.; Saberi, A.; Lai, J.-C.; Wu, Y.; Wang, Y.; Trotsyuk, A. A.; Loh, K. Y.; Shih, C.-C.; Xu, W.; Liang, K.; Zhang, K.; Bai, Y.; Gurusankar, G.; Hu, W.; Jia, W.; Cheng, Z.; Dauskardt, R. H.; Gurtner, G. C.; Tok, J. B.-H.; Deisseroth, K.; Soltesz, I.; Bao, Z. *Science* **2022**, *375*, 1411–1417.

[3] Greschner, A. A.; Brahiti, N.; Auger, M.; Hu, L.; Soleymani Abyaneh, H.; Barbeau, X.; Parent, V.; Gaillet, B.; Guay, D.; Soultan, A.-H.; Gauthier, M. A. *Biomacromolecules* **2023**, *24*, 4890−4900.

[4] Harada, A.; Li, J.; Kamachi, M. *Nature* **1992**, *356*, 325–327.

[5] Schneider, H.-J.; Hacket, F.; Rüdiger, V.; Ikeda, H. *Chem. Rev.* **1998**, *98*, 1755–1785.

[6] Ellis, H. R.; Poole, L. B. *Biochemistry* **1997**, *36*, 15013–15018.

[7] Sun, L.; Bulter, T.; Alcalde, M.; Petrounia, I. P.; Arnold, F. H. *ChemBioChem* **2002**, *3*, 781–783.

[8] Savéant, J.-M. *Chem. Rev.* **2008**, *108*, 2348–2378.

[9] Sohn, Y. S.; Hendrickson, D. N.; Gray, H. B. *J. Am. Chem. Soc.* **1971**, *93*, 3603–3612.

[10] Burns, J. A.; Butler, J. C.; Moran, J.; Whitesides, G. M. *J. Org. Chem.* **1991**, *56*, 2648–2650.
